# Mental health before and during the COVID-19 pandemic in two longitudinal UK population cohorts

**DOI:** 10.1192/bjp.2020.242

**Published:** 2020-11-24

**Authors:** Alex S. F. Kwong, Rebecca M. Pearson, Mark J. Adams, Kate Northstone, Kate Tilling, Daniel Smith, Chloe Fawns-Ritchie, Helen Bould, Naomi Warne, Stanley Zammit, David J. Gunnell, Paul A. Moran, Nadia Micali, Abraham Reichenberg, Matthew Hickman, Dheeraj Rai, Simon Haworth, Archie Campbell, Drew Altschul, Robin Flaig, Andrew M. McIntosh, Deborah A. Lawlor, David Porteous, Nicholas J. Timpson

**Affiliations:** 1 MRC Integrative Epidemiology Unit, University of Bristol, UK; Population Health Sciences, Bristol Medical School, University of Bristol, UK; and Division of Psychiatry, University of Edinburgh, UK; 2 MRC Integrative Epidemiology Unit, University of Bristol, UK; and Population Health Sciences, Bristol Medical School, University of Bristol, UK; 3 Division of Psychiatry, University of Edinburgh, UK; 4 Population Health Sciences, Bristol Medical School, University of Bristol, UK; 5 Department of Psychology, University of Edinburgh, UK; 6 Population Health Sciences, Bristol Medical School, University of Bristol, UK; and Gloucestershire Health and Care NHS Foundation Trust, UK; 7 Population Health Sciences, Bristol Medical School, University of Bristol, UK; and MRC Centre for Neuropsychiatric Genetics and Genomics, Division of Psychological Medicine and Clinical Neurosciences, Cardiff University, UK; 8 Population Health Sciences, Bristol Medical School, University of Bristol, UK; and National Institute of Health Research Biomedical Research Centre, University of Bristol, UK; 9 Population Health Sciences, Bristol Medical School, University of Bristol, UK; National Institute of Health Research Biomedical Research Centre, University of Bristol, UK; and Avon and Wiltshire Mental Health Partnership NHS Trust, UK; 10 Great Ormond Street Institute of Child Health, University College London, UK; Department of Psychiatry, Faculty of Medicine, University of Geneva, Switzerland; and Department of Paediatrics Gynaecology and Obstetrics, Faculty of Medicine, University of Geneva, Switzerland; 11 Department of Psychiatry, Icahn School of Medicine at Mount Sinai, New York, USA; 12 1MRC Integrative Epidemiology Unit, University of Bristol, UK; and Population Health Sciences, Bristol Medical School, University of Bristol, UK; 13 Centre for Genomic and Experimental Medicine, Institute of Genetics & Molecular Medicine, University of Edinburgh, UK; and Usher Institute for Population Health Sciences and Informatics, University of Edinburgh, UK; 14 Centre for Genomic and Experimental Medicine, Institute of Genetics & Molecular Medicine, University of Edinburgh, UK; 15 MRC Integrative Epidemiology Unit at the University of Bristol, UK; Population Health Sciences, Bristol Medical School, University of Bristol, UK; and National Institute of Health Research Biomedical Research Centre, University of Bristol, UK; 16 MRC Integrative Epidemiology Unit at the University of Bristol, UK; and Population Health Sciences, Bristol Medical School, University of Bristol, UK

**Keywords:** COVID-19, ALSPAC, generation Scotland, anxiety disorders, depressive disorders

## Abstract

**Background:**

The COVID-19 pandemic and mitigation measures are likely to have a marked effect on mental health. It is important to use longitudinal data to improve inferences.

**Aims:**

To quantify the prevalence of depression, anxiety and mental well-being before and during the COVID-19 pandemic. Also, to identify groups at risk of depression and/or anxiety during the pandemic.

**Method:**

Data were from the Avon Longitudinal Study of Parents and Children (ALSPAC) index generation (*n* = 2850, mean age 28 years) and parent generation (*n* = 3720, mean age 59 years), and Generation Scotland (*n* = 4233, mean age 59 years). Depression was measured with the Short Mood and Feelings Questionnaire in ALSPAC and the Patient Health Questionnaire-9 in Generation Scotland. Anxiety and mental well-being were measured with the Generalised Anxiety Disorder Assessment-7 and the Short Warwick Edinburgh Mental Wellbeing Scale.

**Results:**

Depression during the pandemic was similar to pre-pandemic levels in the ALSPAC index generation, but those experiencing anxiety had almost doubled, at 24% (95% CI 23–26%) compared with a pre-pandemic level of 13% (95% CI 12–14%). In both studies, anxiety and depression during the pandemic was greater in younger members, women, those with pre-existing mental/physical health conditions and individuals in socioeconomic adversity, even when controlling for pre-pandemic anxiety and depression.

**Conclusions:**

These results provide evidence for increased anxiety in young people that is coincident with the pandemic. Specific groups are at elevated risk of depression and anxiety during the COVID-19 pandemic. This is important for planning current mental health provisions and for long-term impact beyond this pandemic.

## Background

The COVID-19 pandemic has resulted in radical changes to societies globally. As the number of infected cases and deaths increased, many countries adopted public health measures, including lockdown, social distancing, self-isolation and school and business closures, resulting in an unprecedented impact on the global economy, which may also have a profound effect on mental health.^1–3^ However, the extent to which mental health is affected by COVID-19 and its management, and who is at greatest risk, is still unclear. Evidence from previous outbreaks such as the severe acute respiratory syndrome epidemic,^4–8^ and rapid COVID-19 cross-sectional surveys, suggest that depression, anxiety and lower well-being may increase during the COVID-19 pandemic.^9–12^

Several rapid cross-sectional surveys during the pandemic have suggested a higher prevalence of anxiety, depression^9,10^ and low well-being compared with historical estimates.^12^ However, these studies lack pre-pandemic information on mental health assessments, and potential confounding factors, in the same people before the pandemic. This precludes accurate assessment of whether adverse mental health outcomes during the pandemic are largely accounted for by those with existing or previous mental health problems having poorer mental health as a result of COVID-19 mitigation efforts, or whether there are important contributions of the pandemic that are related to poor mental health in those with no previous history. Furthermore, selection bias (because of mental health influencing those who respond to surveys) and reporting bias (from those who perceive depression and anxiety as higher or are more likely to report symptoms when they feel there is a ‘valid’ reason) could threaten the validity of results from cross-sectional surveys. There is a need for longitudinal designs with well-characterised sampling frames and pre-pandemic data.^13^ Such studies can more accurately quantify differences in mental health from pre-pandemic levels, and identify groups that are most at risk of adverse mental health in response to the COVID-19 pandemic. These results can then inform development of interventions for those at heightened risk, and aid policy decisions regarding the immediate and subsequent management of the COVID-19 response. This includes plans for easing restrictions and monitoring at-risk groups as subsequent waves or epidemics occur, and plans for the longer-term care for groups whose mental health may be particularly affected.^1,14^ The COVID-19 pandemic is likely to exacerbate existing social and psychological inequalities.^15^ Previous studies have identified several groups who may be at greater risk of poorer mental health during the pandemic, including younger people, parents, women, healthcare workers and those with poorer financial or living circumstances.^9–11,16^ However, many groups remain unexplored, such as individuals at risk of abuse and those at greater physical risk of COVID-19 (older age, and those with chronic conditions such as asthma or obesity).

## Aims

We aimed to use data from two independent longitudinal cohort studies, both with rich pre-pandemic measures of mental health, to quantify how mental health changed from pre-pandemic levels to the COVID-19 pandemic, and identify groups within the population at greater risk of poorer mental health during the pandemic. The first of these is important for exploring the impact of COVID-19 and its management on mental health and potential increases in poor mental health long term. The second is important for targeting of mental healthcare needs now, and during any subsequent waves, and for identifying groups who might benefit from long-term monitoring after the pandemic.

## Method

### Samples

We selected two comparable cohort studies, to allow replication in different regions of the UK, both with similar timings of mental health measures before and during the COVID-19 pandemic.

The Avon Longitudinal Study of Parents and Children (ALSPAC) is an ongoing longitudinal population-based study that recruited pregnant women residing in Avon (South-West England) with expected delivery dates between 1 April 1991 and 31 December 1992.^17,18^ The cohort comprised 13 761 mothers and their partners (hereafter referred to as ALSPAC-parents), and their 14 901 children, now young adults (hereafter referred to as ALSPAC-young).^19^ The study website contains details of all data, available through a fully searchable data dictionary (http://www.bristol.ac.uk/alspac/researchers/our-data/). Ethical approval for the study was obtained from the ALSPAC Ethics and Law Committee and the Local Research Ethics Committees.

Generation Scotland: Scottish Family Health Study is a family longitudinal study of 24 084 individuals recruited across Scotland between 2006 and 2011.^20^ Participants were recruited into the study if they were aged ≥18 years. Participants of Generation Scotland have been followed up longitudinally,^21^ and further details can be found on the study website (http://www.generationscotland.org). Ethical approval for the study was approved by National Health Service Tayside Committee on Medical Research Ethics (reference 05/S1401/89). We assert that all procedures contributing to this work comply with the ethical standards of the relevant national and institutional committees on human experimentation and with the Helsinki Declaration of 1975, as revised in 2008. Written informed consent was obtained from all participants in both studies.

This study uses data from 3720 individuals in the ALSPAC-parents cohort and 2973 individuals in the ALSPAC-young cohort who completed an online questionnaire about the impact and consequences of the COVID-19 pandemic between 9 April and 14 May 2020 (see Supplementary Figs 1 and 2 available at https://doi.org/10.1192/bjp.2020.242).^22^ In Generation Scotland, data were from 4233 individuals who completed a similar online COVID-19 questionnaire between 17 April and 17 May 2020 (see Supplementary Fig. 3). Mitigation measures were announced in the UK on the 24 March 2020.

### COVID-19 pandemic measures of mental health

The measures used here examine symptoms in the preceding 2 weeks, and thus represent mental health in the immediate period following mitigation. Depressive symptoms in ALSPAC were measured with the Short Mood and Feelings Questionnaire (SMFQ),^23^ a 13-item instrument with scores ranging between 0 and 26, with higher scores indicting higher depressive symptoms. In Generation Scotland, depressive symptoms were measured with the Patient Health Questionnaire-9 (PHQ-9),^24^ a nine-item instrument with scores ranging between 0 and 27, with higher scores indicating worse depressive symptoms. Anxiety symptoms in ALSPAC and Generation Scotland were both measured with the Generalised Anxiety Disorder Assessment-7 (GAD-7),^25^ a seven-item instrument with ranging between 0 and 21, with higher scores indicting greater generalised anxiety disorder symptoms. Mental well-being in ALSPAC and Generation Scotland were both measured with the Short Warwick-Edinburgh Mental Wellbeing Scale (SWEMWBS),^26^ a seven-item instrument with scores ranging between 7 and 35, with higher scores indicating better mental well-being. These measures have recommended cut-offs for examining the proportion of individuals with probable depression (≥11 on the SMFQ and ≥10 on the PHQ-9),^24,27^ generalised anxiety disorder (≥10 on the GAD-7)^28^ and poor mental well-being (≤17 on the SWEMWBS),^29^ with good sensitivity and specificity for identifying clinical disorder, using validated interviews and instruments, and are widely used in primary care and clinical trials. Herein, we refer to depressive symptoms as depression, and anxiety symptoms as anxiety.

### Pre-pandemic assessments of mental health and factors

Pre-pandemic depression and anxiety were assessed in ALSPAC and Generation Scotland before the COVID-19 pandemic. In the ALSPAC-young cohort, pre-pandemic mental well-being was also assessed. The median length of time between the pre-pandemic assessments and COVID-19 pandemic assessments of mental health ranged from 2 to 7 years in the ALSPAC-young cohort, 7 to 20 years in the ALSPAC-parents cohort and 4 to 5 years in the Generation Scotland cohort. These measures, their timings and harmonisation are described in Table 1 and in Supplementary Methods, alongside detailed information on pre-pandemic factors that may be associated with poorer mental health during the COVID-19 pandemic in regression analyses. We refer to these as factors to make it clear that we are not assuming they are causal, but could be useful in the short term, for identifying at-risk groups. Factors included sociodemographic information, such as biological sex, age, educational background, financial circumstances, deprivation status, victimisation and being a parent with school-aged children. Additional factors included pre-existing mental health conditions, substance misuse, genetic risk for depression, cognitive styles, personality traits and difficulties accessing mental health information. Because of differences in data collection, several factors are only assessed in either ALSPAC or Generation Scotland. We also examined associations with several COVID-19-specific factors, including pre-pandemic obesity, pre-pandemic asthma, a self-reported suspected or confirmed diagnosis of COVID-19, isolation status, living alone, access to a garden and healthcare worker and key worker status.

**Table 1.** Pre-pandemic mental health measures and factors assessed in ALSPAC and Generation Scotland

	ALSPAC-parents	ALSPAC-young	Generation Scotland
Sociodemographic factors			
Sex	Questionnaire; 1991–1992	Questionnaire item; 1991–1992	Questionnaire; 2006–2011
Age	Questionnaire; 2020	Questionnaire; 2020	Questionnaire; 2020
Educational background	Questionnaire; 1991–1992	Questionnaire; 1991–1992	Questionnaire; 2006–2011
Income	Questionnaire; 2011–2014	Questionnaire; 2017–2018	Questionnaire; 2006–2011
Deprivation status	Indices of multiple deprivation; 2014	Indices of multiple deprivation; 2014	Indices of multiple deprivation; 2006–2011
Recent financial problems	Questionnaire; 2010–2013	Questionnaire; 2018–2020	Questionnaire; 2015–2016
Partner emotional abuse	Life Events Questionnaire; 2010–2013	Not assessed	Not assessed
Parent with young children	Not assessed	Questionnaire; 2012–2020	Questionnaire; 2020
Physical health factors			
Obesity	BMI > 30 kg/m^2^, assessed at a research clinic; 2011–2015	BMI > 30 kg/m^2^, assessed at a research clinic; 2015–2017	BMI > 30 kg/m^2^, assessed at a research clinic; 2006–2011
Asthma	Questionnaire; 2002–2004	Questionnaire; 2014–2015	Questionnaire; 2006–2011
COVID-19-specific factors			
Infection status	Questionnaire; 2020	Questionnaire; 2020	Questionnaire; 2020
Isolation status	Questionnaire; 2020	Questionnaire; 2020	Not assessed
Living alone	Questionnaire; 2020	Questionnaire; 2020	Questionnaire; 2020
No access to a garden	Questionnaire; 2020	Questionnaire; 2020	Questionnaire; 2020
Healthcare worker	Questionnaire; 2020	Questionnaire; 2020	Not assessed
Key worker	Questionnaire; 2020	Questionnaire; 2020	Questionnaire; 2020
Pre-pandemic mental health measures			
Depressive symptoms	Edinburgh Postnatal Depression Scale^30^; 2011–2013	Short Mood and Feelings Questionnaire^23^; 2017–2018	General Health Questionnaire – Depression^31^; 2015–2016
Anxiety	Spielberger Trait Anxiety Inventory^32^; 1999–2001	Generalised Anxiety Disorder Assessment^25^;^a^ 2014–2015	General Health Questionnaire – Anxiety^31^; 2015–2016
Mental well-being	Not assessed	Short Warwick-Edinburgh Mental Wellbeing Scale^26^; 2015–2016	Not assessed
Psychiatric or mental health factors			
Probable major depression	Life Events Questionnaire; 2001–2003	Clinical Interview Schedule – Revised^33^; 2015–2017	Structured Clinical Interview for DSM-IV^34^; 2006–2011
Probable generalised anxiety disorder	Life Events Questionnaire; 2003–2005	Clinical Interview Schedule – Revised^33^; 2015–2017	Not assessed
Psychosis-like experiences	Not assessed	Psychosis-Like Symptoms Interview^35^; 2015–2017	Schizotypal Personality Questionnaire^36^; 2006–2011
Disordered eating	Life Events Questionnaire; 2001–2003	Questionnaire consistent with DSM-V frequency; 2016–2017	Not assessed
Obsessive–compulsive disorder traits	Not assessed	Obsessive–Compulsive Inventory^37^; 2016–2017	Not assessed
Autistic traits	Not assessed	Social Responsiveness Scale^38^; 2016–2017	Not assessed
Personality disorder traits	Karolinska Scales of Personality^39^; 2000–2002	Standardised Assessment of Personality: Abbreviated Scale^40^; 2015–2017	Not assessed
History of alcohol misuse	Alcohol Use Disorders Identification Test^41^; 2011–2013	Alcohol Use Disorders Identification Test^41^; 2015–2017	Questionnaire; 2006–2011
Current smokers (tobacco)	Questionnaire; 2011–2013	Questionnaire; 2015–2017	Questionnaire; 2006–2011
Cognitive styles	Negative Schemas Questionnaire^42^, 2011–2013	Cognitive Styles Questionnaire^43^; 2008–2010	Brief Resilience Scale^44^; 2015–2016
Difficulties accessing mental health information	Not assessed	Questionnaire; 2017–2018	Not assessed
Neuroticism	Not assessed	Big Five Factors of Personality – Neuroticism^45^; 2005–2006	Eysenck Personality Questionnaire – Neuroticism^46^; 2006–2011
Self-harm history	Not assessed	Questionnaire; 2015–2017	Linkage to lifetime medical records
Depression polygenic risk score	Constructed from a recent GWAS on depression^47^	Constructed from a recent GWAS on depression^47^	Constructed from a recent GWAS on depression^47^

Data are shown in format: measure used in analyses; timing of assessment. Questionnaire refers to a single questionnaire item used. ALSPAC-parents, original parents in the Avon Longitudinal Study of Parents and Children; ALSPAC-young, original children in the Avon Longitudinal Study of Parents and Children; BMI, body mass index; GWAS, genome-wide association study.

a. We supplemented data with more recent timings of anxiety symptoms from the Clinical Interview Schedule – Revised between 2015 and 2017.

### Statistical analysis

Analysis was conducted in StataSE, version 15 for Windows(StataCorp LLC). Initially, we described the prevalence of mental health outcomes during the COVID-19 pandemic in all cohorts. To answer our first research objective (How has the prevalence of mental health changed from pre-pandemic to COVID-19?), we used the ALSPAC-young cohort to quantify differences in mental health from pre-pandemic to COVID-19 levels, as this was the only cohort with the identical measures of mental health assessed before and during the pandemic (i.e., the SMFQ, GAD-7 and SWEMWBS). Our hypothesis is that the COVID-19 pandemic is likely to have caused a rise in depression and anxiety in the population as a whole. This hypothesis cannot be explicitly tested, given that COVID-19 mitigation efforts have been a universal exposure. However, looking at changes from baseline analysis provides some initial evidence for this hypothesis. The GAD-7 was only assessed once before the pandemic (between 2014 and 2015), so we also compared the prevalence of anxiety during the COVID-19 pandemic with probable anxiety disorder as assessed with the Clinical Interview Schedule – Revised,^33^ at two other occasions (between 2008 and 2010 and between 2015 and 2017). This was to provide more thorough pre-pandemic information on anxiety, and to harmonise timings for pre-pandemic mental health. We also analysed item-level responses for the SMFQ, GAD-7 and SWEMWBS to examine how specific mental health items differed before and during the pandemic.

To answer our second research objective (Are there groups within the general population that are at heightened risk of depression and anxiety during the COVID-19 pandemic?), we examined associations between factors measured before and during the pandemic with depression and anxiety during the COVID-19 pandemic, accounting for pre-pandemic depression and anxiety (with the most recent matched measures available). We included pre-pandemic measures of depression and anxiety as covariates into our regression models, so the shared variance between pre-pandemic and COVD-19 pandemic depression and anxiety were accounted for. The adjusted coefficients represent the extent to which the factors are associated with depression and anxiety during the COVID-19 pandemic, independent of prior mental health. Analysis was conducted separately for all cohorts, adjusted for sex, age and the date they completed the COVID-19 questionnaire (to account for response times). We chose to run minimised regressions to avoid potential biases from collider bias and to identify risk populations who could be followed up in greater detail, with more specific confounding structures. Pre-pandemic mental well-being was not assessed in ALSPAC-parents or Generation Scotland cohorts; therefore, we restricted this analysis to depression and anxiety only. Continuous COVID-19 pandemic and pre-pandemic depression and anxiety were standardised to create *Z*-scores, allowing comparison of effect sizes across outcomes and cohorts.

#### Missing data

Our eligible samples were those who completed at least one mental health measure during the COVID-19 surveys: ALSPAC-parents cohort, *n* = 3579; ALSPAC-young cohort, *n* = 2872 and Generation Scotland cohort, *n* = 4208 (Supplement Figs 1–3, Supplement Table 1). To address potential bias from attrition in the cohorts, we imputed pre-pandemic depression, anxiety and factors, with related information and auxiliary variables up to the eligible samples, using multiple imputation by chained equations to generate 50 imputed data-sets.^48^ Details regarding imputation and a list of auxiliary variables are available in the Supplementary Material.

#### Sensitivity analyses

We ran several sensitivity analyses, including complete-case analysis, adjusting for educational background with the imputed data, using the validated cut-offs rather than continuous scores as outcomes and using varying timings for pre-pandemic depression, anxiety and financial problems, to ensure there were no substantial changes as a result of proximal timings between assessments and the COVID-19 pandemic. We also estimated ‘counterfactual’ trajectories for the mental health measures in the ALSPAC-young cohort to highlight differences in what would we expected had COVID-19 not happened, given previous trajectories, compared with what was observed during the pandemic. Finally, we analysed item-level responses for the SMFQ, GAD-7 and SWEMWBS to examine how specific components of each measurement differed from the most recent pre-pandemic assessment and the COVID-19 pandemic assessment. Further information regarding these analyses are given in the Supplementary Material.

## Results

Data on mental health outcomes during the COVID-19 pandemic were available for 3579 people (mean age 58.67 years, s.d. 4.82) for the ALSPAC-parents cohort, 2872 people (mean age 27.61 years, s.d. 0.54) for the ALSPAC-young cohort and 4208 people (mean age 59.24 years, s.d. 12.03) for the Generation Scotland cohort. Individuals included in these analyses were more likely to be female and have higher educational backgrounds, but did not differ by pre-existing depression or anxiety symptoms (Supplementary Table 1).

### Prevalence of mental health outcomes during the COVID-19 pandemic

The prevalence of probable depression during the COVID-19 pandemic was highest for younger individuals (ages 18–40 years), and decreased with older age in ALSPAC and Generation Scotland. Similar results were observed for probable anxiety and lower well-being (Supplementary Figs 4 and 5).

### Differences in mental health before and during the COVID-19 pandemic in the ALSPAC-young cohort

**Fig. 1:**
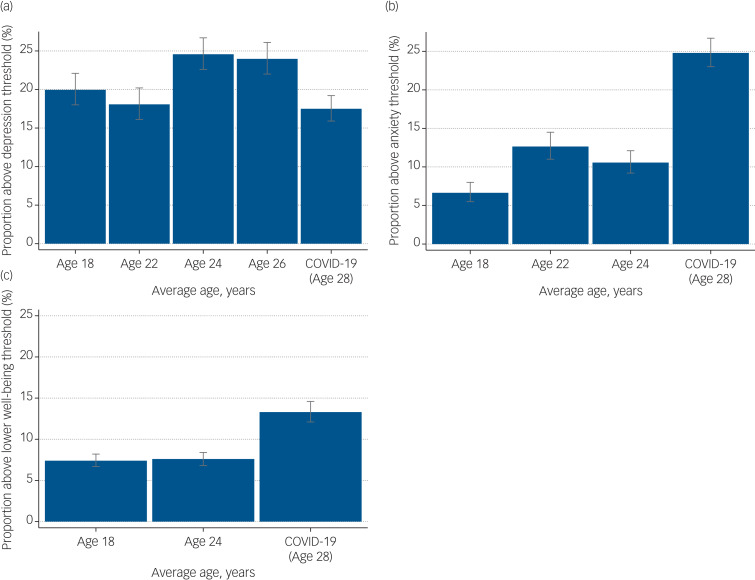
Changes in mental health from pre-pandemic to during the COVID-19 pandemic in the ALSPAC-young cohort. (a) Changes in probable depression, as assessed by the SMFQ. (b) Changes in probable generalised anxiety disorder, as assessed by the GAD-7 at age 22 years and the CISR GAD at ages 18 and 24 years. (c) Changes in lower well-being, as assessed by the SWEMWBS. ALSPAC-young, original children in the Avon Longitduinal Study of Parents and Children; CISR GAD, Clinical Interview Schedule – Revised, Generalised Anxiety Disorder; GAD-7, Generalised Anixety Disorder Assessment; SMFQ, Short Mood and Feelings Questionnaire; SWEMWBS, Short Warwick Edinburgh Mental Wellbeing Scale.

The percentage of ALSPAC-young participants with probable depression was lower during the pandemic, at 18.14% (95% CI 16.76–19.61%) compared with 24.35% (95% CI 23.04–26.70%) at the most recent pre-pandemic assessment. However, the percentage of participants with probable anxiety disorder almost doubled during the pandemic, at 24.35% (95% CI 22.81–25.96%) compared with pre-pandemic levels (12.97%, 95% CI 11.87–14.15%); as did the percentage for lower well-being, at 13.27% (95% CI 12.07–14.15%) compared with 7.59% (95% CI 6.82–8.43%) (Fig. 1, Supplementary Tables 5 and 6). When examining continuous measures for comparisons between the most recent pre-pandemic and pandemic assessments, there was a mean difference of −0.60 (95% CI −0.84 to −0.37) in SMFQ score, 1.36 (95% CI 1.10–1.61) in GAD-7 score and 2.45 (95% CI 2.25–2.65) in SWEMWBS score. For magnitude, these estimates represent a 0.11, 0.26 and 0.51 standardised effect difference for depression, anxiety and lower well-being, respectively (Supplementary Table 7). Item-level analysis showed that for the SMFQ (depression), the only items that scored higher during the COVID-19 pandemic were ‘no enjoyment’, ‘felt restless’ and ‘found it hard to think’. For the GAD-7 (anxiety) and the SWEMWBS (mental well-being), all items scored higher during the COVID-19 pandemic compared with the most recent pre-pandemic assessment, with similar variances (Supplementary Fig. 6).

### Factors related to depression and anxiety during the COVID-19 pandemic

**Fig. 2:**
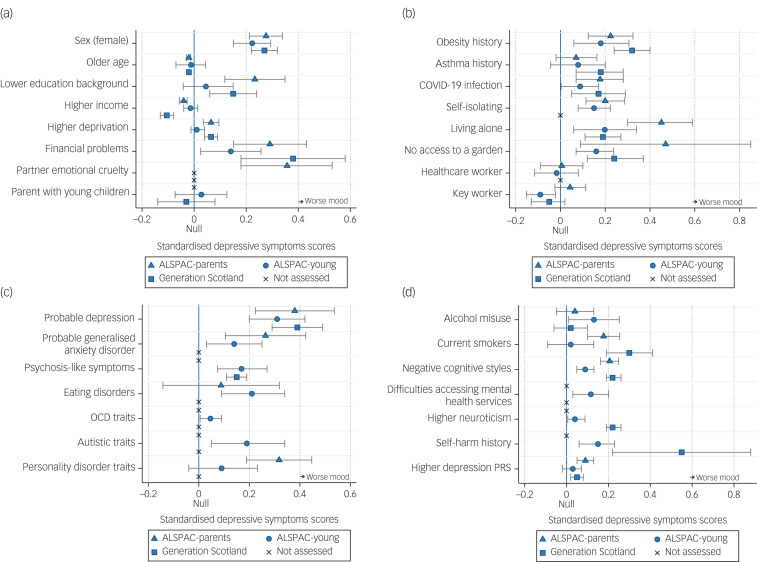
Associations between pre-pandemic and COVID-19-specific factors and depression during the COVID-19 pandemic, adjusted for the most recent pre-pandemic assessment of depression, sex, age and when the COVID-19 questionnaire was completed, using imputed data (estimates match Supplementary Table 8). Estimates refer to an s.d. increase in depression, over and above pre-pandemic depression. (a) Associations between pre-pandemic sociodemographic factors and depression during the COVID-19 pandemic. (b) Associations between pre-pandemic physical health and COVID-19-specific factors and depression during the COVID-19 pandemic. (c and d) Associations between pre-pandemic mental health factors and depression during the COVID-19 pandemic. ALSPAC-parents, original parents in the Avon Longitudinal Study of Parents and Children; ALSPAC-young, original children in the Avon Longitudinal Study of Parents and Children; OCD, obsessive compulsive disorder; PRS, polygenic risk score.

**Fig. 3:**
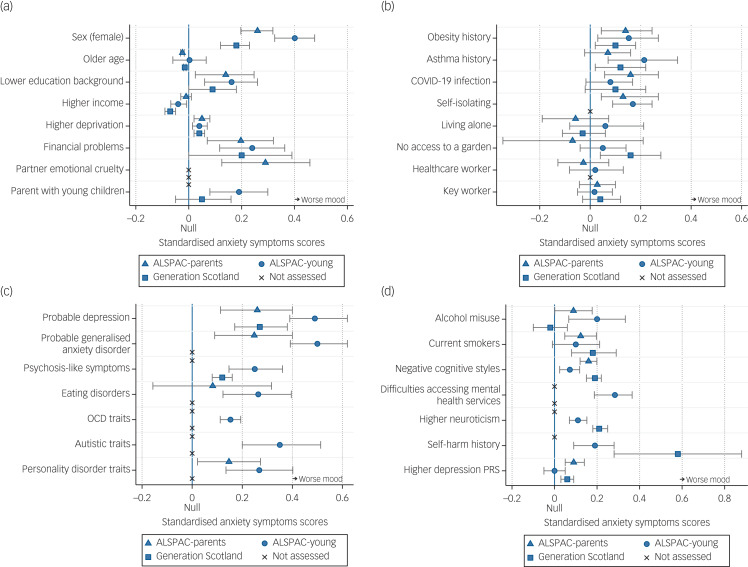
Associations between pre-pandemic and COVID-19-specific factors and anxiety during the COVID-19 pandemic, adjusted for the most recent pre-pandemic assessments of anxiety, sex, age and when the COVID-19 questionnaire was completed, using imputed data (estimates match Supplementary Table 8). Estimates refer to an s.d. increase in anxiety, over and above pre-pandemic anxiety. (a) Associations between pre-pandemic sociodemographic factors and anxiety during the COVID-19 pandemic. (b) Associations between pre-pandemic physical health and COVID-19-specific factors and anxiety during the COVID-19 pandemic. (c and d) Associations between pre-pandemic mental health factors and anxiety during the COVID-19 pandemic. ALSPAC-parents, original parents in the Avon Longitudinal Study of Parents and Children; ALSPAC-young, orignal children in the Avon Longitudinal Study of Parents and Children; OCD, obsessive compulsive disorder; PRS, polygenic risk score.

Supplementary Table 8 and Figs 2 and 3 show all the associations between pre-pandemic and COVID-19-specific factors and depression and anxiety during the early stages of COVID-19, accounting for pre-pandemic assessments of depression and anxiety (measured on a continuous scale in s.d. units for ease of comparison between cohorts and outcomes, i.e. standardised regression coefficients). As stated above, these results can be interpreted as factors associated with depression and anxiety during the pandemic, after accounting for previous depression and anxiety, thus resulting in associations that are independent of prior mental health.

### Pre-pandemic sociodemographic factors

Being female and pre-pandemic financial problems were associated with higher depression and anxiety during the COVID-19 pandemic in ALSPAC-parents, ALSPAC-young and Generation Scotland cohorts. Lower educational background was associated with greater depression in ALSPAC-parents and Generation Scotland cohorts, but not in the ALSPAC-young cohort; and with greater anxiety in ALSPAC-parents and ALSPAC-young cohorts, but not in the Generation Scotland cohort. Higher income before the pandemic was associated with lower depression in ALSPAC-parents and Generation Scotland cohorts, but not in the ALSPAC-young cohort; and with lower anxiety in ALSPAC-young and Generation Scotland cohorts, but not in the ALSPAC-parents cohorts. Higher neighbourhood deprivation was associated with higher depression in ALSPAC-parents and Generation Scotland cohorts, but not in the ALSPAC-young cohort; and with higher anxiety in ALSPAC-parents, ALSPAC-young and Generation Scotland cohorts. Being a parent of a young child was associated with higher anxiety in the ALSPAC-young cohort, but not in the Generation Scotland cohort (not assessed in the ALSPAC-parents cohort), and was not associated with depression in either cohort. Reporting an emotionally abusive partner was only available in the ALSPAC-parents cohort, and was positively associated with both greater depression and anxiety. Estimates are given in Supplementary Table 8 and shown in Figs 2A and 3A.

### Physical health factors

Pre-pandemic obesity was associated with higher depression and anxiety in all cohorts. Pre-pandemic asthma status had positive associations with higher anxiety in ALSPAC-young and Generation Scotland cohorts, but not in the ALSPAC-parents cohort; and with higher depression in the Generation Scotland cohort, but not in either ALSPAC cohorts (see Supplementary Table 8 and Figs 2B and 3B).

### COVID-19-specific factors

A self-reported suspected or confirmed COVID-19 diagnosis was associated with higher depression and anxiety in the ALSPAC-parents cohort, but only higher depression in Generation Scotland and ALSPAC-young cohorts. Living alone during the pandemic was also associated with higher depression in all cohorts, but was not associated with anxiety. No access to a garden was associated with higher depression in ALSPAC-parents, ALSPAC-young and Generation Scotland cohorts, along with higher anxiety in the Generation Scotland cohort. Self-isolation was associated with higher depression and anxiety in both ALSPAC-parents and ALSPAC-young cohorts, but was not measured in the Generation Scotland cohort. Key workers (of any kind) and healthcare workers were not associated with higher depression or anxiety in any cohort. However, there was an association between being a key worker and lower depression in the ALSPAC-young cohort, but this was not replicated in the ALSPAC-parents or Generation Scotland cohorts. Estimates are given in Supplementary Table 8 and shown in Figs 2B and 3B.

### Pre-pandemic mental health and psychological factors

There were consistent associations for factors such as a history of major depression, a history of generalised anxiety disorder (not assessed in Generation Scotland), negative cognitive styles, psychosis-like symptoms, higher neuroticism and a history of self-harm (latter three not assessed in the ALSPAC-parents cohort). Eating disorder traits were associated with higher depression and anxiety in the ALSPAC-young cohort, but not in the ALSPAC-parents cohort, and was not assessed in Generation Scotland. Personality disorder traits were associated with greater depression in the ALSPAC-parents cohort, but not in the ALSPAC-young cohort; and with higher anxiety in both ALSPAC cohorts, but was not assessed in Generation Scotland. The depression polygenic risk score was positively associated with depression and anxiety in ALSPAC-parents and Generation Scotland cohorts, but was not associated with either depression or anxiety in the ALSPAC-young cohort. A history of alcohol misuse was associated with increased depression in the ALSPAC-young cohort, but not in ALSPAC-parents or Generation Scotland cohorts; and with increased anxiety in ALSPAC-parents and ALSPAC-young cohorts, but not in the Generation Scotland cohort. By contrast, daily smoking was associated with increased depression and anxiety in ALSPAC-parents and Generation Scotland cohorts, but not in the ALSPAC-young cohort. Several factors only measured in the ALSPAC-young cohort showed strong associations with higher depression and anxiety during the COVID-19 pandemic, including high obsessive–compulsive traits, high autistic traits and pre-pandemic reporting of difficulties accessing mental health services (see Supplementary Table 8 and Figs 2C, 2D, 3C and 3D).

### Sensitivity analyses

The main results were similar to the complete-case analysis (Supplementary Table 9), adjusting for educational background with the imputed sample (Supplementary Table 10); examining different pre-pandemic timings of depression, anxiety and factors (Supplementary Tables 11–13); and examining the binary outcomes of depression and anxiety (Supplementary Table 14). Trajectory analyses (Supplementary Fig. 7) suggested higher anxiety and lower well-being during the COVID-19 pandemic were not expected, given the previous trajectory of pre-pandemic assessments. However, depression was in line with expectations.

## Discussion

We report a population-based longitudinal study examining mental health during the COVID-19 pandemic. Although we found no clear evidence that depression differed during the COVID-19 pandemic from pre-pandemic assessments, there was strong observational evidence that anxiety was higher and well-being was lower during the pandemic, compared with pre-pandemic levels. Irrespective of the overall population differences in depression and anxiety, several sociodemographic, psychological, physical and COVID-19-specific factors were associated with greater depression and anxiety during the COVID-19 pandemic.

### Mental health during the COVID-19 pandemic compared with pre-pandemic assessments

Approximately twice as many young adults experienced probable anxiety disorder and lower well-being during the pandemic, compared with previous longitudinal assessments. The mean rises of 0.26 s.d. in GAD-7 scores and 0.51 s.d. in SWEMWBS scores represent effect sizes that are clinically important and of a magnitude similar to those seen following treatment.^49,50^ The rise in magnitude of anxiety and reduction in well-being in the ALSPAC-young cohort goes against expectations in the absence of COVID-19, highlighted in our sensitivity analysis with counterfactual trajectories, and shown by the wealth of descriptive mental health data in ALSPAC. The uncertainty and sudden change to everyday life, as well as concerns over health, may explain why anxiety has initially risen, rather than depression. The apparent rise in younger ages may reflect the effects of mitigation measures (i.e. lockdown and social distancing) rather than a risk of COVID-19 infection (potentially higher in older populations). Furthermore, depression usually relates to feelings of loss,^51^ whereas anxiety relates to threat,^52^ which in this case could be rapid change in society and potential for adverse social and psychological outcomes. There is also evidence that anxiety changes more rapidly than depression after treatment.^49^ What separates this pandemic from historical outbreaks is the global impact. This, alongside the community spirit, may have been initially protective against the self-blame and guilt intrinsic to depression.^51^ Indeed, the depression items that scored lower in the pandemic, compared with pre-pandemic assessments, related to feelings of self-blame. However, this may change as the pandemic evolves and restrictions are eased, thus continued monitoring of mental health is vital for understanding both the short- and long-term impact of the pandemic.

### Population factors associated with depression and anxiety during the COVID-19 pandemic

When accounting for pre-pandemic depression and anxiety, a reported or suspected COVID-19 infection was a factor for higher depression and anxiety during the pandemic in ALSPAC-parents and Generation Scotland cohorts, possibly reflecting the high perceived risk to physical health in older ages and supporting previous research,^2^ but must be interpreted with some caution because COVID-19 status in this study largely includes participants’ perception that they have COVID-19 (because of a lack of testing at this stage of the pandemic). It may be that those with pre-existing depression and anxiety are also more likely to perceive that their symptoms are COVID-19-related, and are therefore subject to reverse causation. There was consistent evidence from participants in ALSPAC and Generation Scotland that health risk groups previously associated with COVID-19, such as those with pre-pandemic obesity and, to some extent, pre-pandemic asthma, had higher depression and anxiety during the pandemic, potentially reflecting concerns regarding perceived risk of infection or the effects of more stringent social distancing. There was no evidence that key workers or health workers were at greater risk of depression or anxiety, suggesting that these groups are not yet experiencing difficulties.

Those who were self-isolating were at higher risk of both anxiety and depression, but living alone was consistently associated with greater depression only. The manifestation of depression rather than anxiety for those living alone may relate to loneliness, which is amplified with physical contact restricted to within households, again reflecting depression being related to absence and loss rather than threat, whereas self-isolation (which in this context is related to COVID-19 exposure) may be linked to anxiety through associated threat of the virus. Parents of young children were more anxious in ALSPAC, which may reflect stress related to the sudden change in child care provision. Financial problems, lower income and deprivation were associated with greater risk of depression and anxiety in ALSPAC-parents and Generation Scotland cohorts. Financial problems were also associated with higher depression and anxiety in the ALSPAC-young cohort. Although these cohorts may have different populations, the replication of financial concerns highlights the importance of global policies to mitigate the sudden socioeconomic impact of the pandemic. and ensure financial measures are accessible to those in need.^53^

As expected, individuals with a history of worse mental health across multiple domains were at greater risk of higher depression and anxiety during the pandemic, supporting concerns raised at the beginning of this pandemic^1,15,54,55^ and highlighting groups who could benefit from immediate support. Personality traits such as neuroticism and negative thinking patterns were strong factors for higher depression and anxiety during the pandemic, and are modifiable with interventions that could benefit those at risk currently or in future outbreaks, even if delivered remotely.^56^

However, there are several limitations. First, as the pandemic is a universal exposure, it is difficult to attribute COVID-19 or its management directly to mental health outcomes. Many factors are likely to show an association with later depression and anxiety at any time.^51^ However, we were able to use rich longitudinal data and methods before and during the COVID-19 pandemic, to demonstrate that anxiety and lower well-being were worse during the pandemic compared with several recent occasions. Sensitivity analysis suggested that this may go against expectations, but detailed follow-up of these measures is essential to fully understand the trajectory of mental health as a result of the COVID-19 pandemic. Nevertheless, it is likely these effects are, to some extent, related to COVID-19, and these results are some of the first to highlight observational associations that could be tested in more causal settings in future. Second, there were some differences in the measures used to assess mental health, both in the pre-pandemic and COVID-19 surveys and across cohorts. The ALSPAC-young cohort was the only cohort that had the same measures of mental health before and during the pandemic, meaning we were only able to accurately describe the change in mental health in this cohort. Thus, the substantial increase in anxiety and decrease in well-being may only be relevant to young populations. Caution must be taken when comparing change over time in the ALSPAC-parents cohort analysis because the measures differed before and during the pandemic. However, our analysis exploring the relationship between factors and mental health during the COVID-19 pandemic remains valid, as despite different pre-pandemic and COVID-19 pandemic measures, the underlying constructs of the measures are the same. These measures were validated against the same standard interview measures, meaning that it is possible to compare longitudinal associations across cohorts, a major strength of our study compared with some previous cross-sectional research. A third limitation is the difference in follow-up length across cohorts. These can be subject to recency effects, where results are stronger if they were measured more proximal to the outcome. However, sensitivity analyses exploring different pre-pandemic timings for depression and anxiety as mental health covariates, and for financial problems as a factor, gave similar conclusions, as shown in Supplementary Tables 11–13. Furthermore, under usual circumstances, depression and anxiety in adulthood are relatively stable, with measures several years apart showing high correlations.^57,58^ Thus, even measures assessed a number of years ago are valid methods to account for previous mental health. Finally, although we were able to use existing data such as educational background to identify pre-pandemic missingness and use such variables in imputation models, we did not impute any data beyond the sample with complete COVID-19 survey data, given that the data were unique. Thus, there may be issues with generalisability, as respondents were more likely to be female and from higher educational backgrounds than previous surveys.

In conclusion, these results are some of the first to provide an initial indication that the COVID-19 pandemic and related mitigation measures are associated with a clinically relevant increase in anxiety in younger populations. Several groups within the population were at heightened risk of higher levels of depression and anxiety during the pandemic. Future work is needed to understand the mechanisms and interplay between pre-pandemic and COVID-19-specific factors and mental health during the COVID-19 pandemic. Future research should consider how changes in anxiety might influence public behaviour through contact patterns and adherence to policies. Depression and anxiety, along with associated impairment, should continue to be carefully monitored to forecast the long-term impact of this crisis. This can help to ensure that future policies consider optimal preservation of both physical and mental health.

## Data Availability

ALSPAC data is available to researchers through an online proposal system. Information regarding access can be found on the ALSPAC website (http://www.bristol.ac.uk/media-library/sites/alspac/documents/researchers/data-access/ALSPAC_Access_Policy.pdf). Generation Scotland: Scottish Family Health Study data is available to researchers on application to the Generation Scotland Access Committee (access@generationscotland.org). The managed access process ensures that approval is granted only to research that comes under the terms of participant consent.

## Supplementary material

For supplementary material accompanying this paper visit https://doi.org/10.1192/bjp.2020.24. click here to view supplementary material

## References

[1] Holmes EA, O'Connor RC, Perry VH, Tracey I, Wessely S, Arseneault L, Multidisciplinary research priorities for the COVID-19 pandemic: a call for action for mental health science. Lancet Psychiatry 2020; 7: 547–60. 32304649 10.1016/S2215-0366(20)30168-1 PMC7159850

[2] Rogers JP, Chesney E, Oliver D, Pollak TA, McGuire P, Fusar-Poli P, Psychiatric and neuropsychiatric presentations associated with severe coronavirus infections: a systematic review and meta-analysis with comparison to the COVID-19 pandemic. Lancet Psychiatry 2020; 7: 611–27. 32437679 10.1016/S2215-0366(20)30203-0 PMC7234781

[3] Brooks SK, Webster RK, Smith LE, Woodland L, Wessely S, Greenberg N, The psychological impact of quarantine and how to reduce it: rapid review of the evidence. Lancet 2020; 395(10227): 912–20. 32112714 10.1016/S0140-6736(20)30460-8 PMC7158942

[4] Lam MH, Wing Y, Yu W, Leung C, Ma RCW, Kong APS, Mental morbidities and chronic fatigue in severe acute respiratory syndrome survivors: long-term follow-up. Arch Intern Med 2009; 169(22): 2142–7. 20008700 10.1001/archinternmed.2009.384

[5] Cheung YT, Chau PH, Yip PS. A revisit on older adults suicides and severe acute respiratory syndrome (SARS) epidemic in Hong Kong. Int J Geriatr Psychiatry 2008; 23(12): 1231–8. 18500689 10.1002/gps.2056

[6] Mak IW, Chu CM, Pan PC, Yiu MG, Chan VL Long-term psychiatric morbidities among SARS survivors. Gen Hosp Psychiatry 2009; 31(4): 318–26. 19555791 10.1016/j.genhosppsych.2009.03.001 PMC7112501

[7] Lau JTF, Griffiths S, Choi KC, Tsui HY Avoidance behaviors and negative psychological responses in the general population in the initial stage of the H1N1 pandemic in Hong Kong. BMC Infect Dis 2010; 10: 139. 10.1186/1471-2334-10-139 PMC2891756 20509887

[8] Tsang HWH, Scudds RJ, Chan EYL. Psychosocial impact of SARS. Emerg Infect Dis 2004; 10(7): 1326–7. 15338536 10.3201/eid1007.040090 PMC3323309

[9] Qiu J, Shen B, Zhao M, Wang Z, Xie B, Xu Y A nationwide survey of psychological distress among Chinese people in the COVID-19 epidemic: implications and policy recommendations. Gen Psychiatr 2020; 33(2): e100213. 32215365 10.1136/gpsych-2020-100213 PMC7061893

[10] Lai J, Ma S, Wang Y, Cai Z, Hu J, Wei N, Factors associated with mental health outcomes among health care workers exposed to coronavirus disease 2019. JAMA Netw Open 2020; 3(3): e203976. 32202646 10.1001/jamanetworkopen.2020.3976 PMC7090843

[11] Fancourt D, Bu F, Mak HW, Steptoe A. *COVID-19 Social Study - Results Release 10* [30 May 2020]. 2020 (https://www.covidsocialstudy.org/results).

[12] Office for National Statistics (ONS). *Personal and Economic Well-Being in Great Britain: May 2020* ONS, 2020 (https://www.ons.gov.uk/peoplepopulationandcommunity/wellbeing/bulletins/personalandeconomicwellbeingintheuk/may2020).

[13] Pierce M, McManus S, Jessop C, John A, Hotopf M, Ford T, Says who? The significance of sampling in mental health surveys during COVID-19. Lancet Psychiatry 2020; 7: 567–8. 32502467 10.1016/S2215-0366(20)30237-6 PMC7266586

[14] Xiang Y-T, Yang Y, Li W, Zhang L, Zhang Q, Cheung T, Timely mental health care for the 2019 novel coronavirus outbreak is urgently needed. Lancet Psychiatry 2020; 7(3): 228–9. 32032543 10.1016/S2215-0366(20)30046-8 PMC7128153

[15] Gunnell D, Appleby L, Arensman E, Hawton K, John A, Kapur N, Suicide risk and prevention during the COVID-19 pandemic. Lancet Psychiatry 2020; 7: 468–71. 32330430 10.1016/S2215-0366(20)30171-1 PMC7173821

[16] McGinty EE, Presskreischer R, Han H, Barry CL. Psychological distress and loneliness reported by US adults in 2018 and April 2020. JAMA 2020; 324: 93–4. 32492088 10.1001/jama.2020.9740 PMC7270868

[17] Boyd A, Golding J, Macleod J, Lawlor DA, Fraser A, Henderson J, Cohort profile: the ‘children of the 90s'–the index offspring of the Avon Longitudinal Study of Parents and Children. Int J Epidemiol 2013; 42(1): 111–27. 22507743 10.1093/ije/dys064 PMC3600618

[18] Fraser A, Macdonald-Wallis C, Tilling K, Body A, Golding J, Davey Smith G, Cohort profile: the Avon Longitudinal Study of Parents and Children: ALSPAC mothers cohort. Int J Epidemiol 2013; 42(1): 97–110. 22507742 10.1093/ije/dys066 PMC3600619

[19] Northstone K, Lewcock M, Groom A, Boyd A, Macleod J, Timpson N, The Avon Longitudinal Study of Parents and Children (ALSPAC): an update on the enrolled sample of index children in 2019. Wellcome Open Res 2019; 4: 51. 31020050 10.12688/wellcomeopenres.15132.1 PMC6464058

[20] Smith BH, Campbell A, Linksted P, Fitzpatrick B, Jackson C, Kerr SM, Cohort profile: Generation Scotland: Scottish Family Health Study (GS:SFHS). The study, its participants and their potential for genetic research on health and illness. Int J Epidemiol 2013; 42(3): 689–700. 22786799 10.1093/ije/dys084

[21] Navrady LB, Wolters MK, MacIntyre DJ, Clarke TK, Campbell AI, Murray AD, Cohort profile: stratifying resilience and depression longitudinally (STRADL): a questionnaire follow-up of Generation Scotland: Scottish Family Health Study (GS:SFHS). Int J Epidemiol 2018; 47(1): 13–14g. 29040551 10.1093/ije/dyx115 PMC5837716

[22] Northstone K, Haworth S, Smith D, Bowring C, Wells N, Timpson NJ. The Avon Longitudinal Study of Parents and Children - a resource for COVID-19 research: questionnaire data capture April-May 2020. Wellcome Open Res 2020; 5: 210. 32995559 10.12688/wellcomeopenres.16225.1 PMC7512032

[23] Angold A, Costello EJ, Messer SC, Pickles A. Development of a short questionnaire for use in epidemiological studies of depression in children and adolescents. Int J Methods Psychiatr Res 1995; 5(4): 237–49.

[24] Kroenke K, Spitzer RL, Williams JB. The PHQ-9: validity of a brief depression severity measure. J Gen Intern Med 2001; 16(9): 606–13. 11556941 10.1046/j.1525-1497.2001.016009606.x PMC1495268

[25] Spitzer RL, Kroenke K, Williams JBW, Lowe B. A brief measure for assessing generalized anxiety disorder. Arch Intern Med 2006; 166: 1092–7. 16717171 10.1001/archinte.166.10.1092

[26] Stewart-Brown S, Tennant A, Tennant R, Platt S, Parkinson J, Weich S. Internal construct validity of the Warwick-Edinburgh Mental Well-being Scale (WEMWBS): a Rasch analysis using data from the Scottish Health Education Population Survey. Health Qual Life Outcomes 2009; 7: 15. 19228398 10.1186/1477-7525-7-15 PMC2669062

[27] Turner N, Joinson C, Peters TJ, Wiles N, Lewis G. Validity of the Short Mood and Feelings Questionnaire in late adolescence. Psychol Assess 2014; 26: 752–62. 24749755 10.1037/a0036572

[28] Kroenke K, Spitzer RL, Williams JBW, Monahan PO, Lowe B. Anxiety disorders in primary care prevalence, impairment, comorbidity, and detection. Ann Intern Medicine 2007; 146: 317–25. 10.7326/0003-4819-146-5-200703060-00004 17339617

[29] Warwick Medical School. *Collect, Score, Analyse and Interpret WEMWBS.* University of Warwick, 2020 (https://warwick.ac.uk/fac/sci/med/research/platform/wemwbs/using/howto/).

[30] Cox JL, Holden JM, Sagovsky R. Detection of postnatal depression: development of the 10-item Edinburgh Postnatal Depression Scale. Br J Psychiatry 1987; 150: 782–6. 3651732 10.1192/bjp.150.6.782

[31] Goldberg D. General Health Questionnaire. NFER Publishing Company, 1978.

[32] Spielberger CD, Gorssuch RL, Lushene PR, Vagg PR, Jacobs GA. Manual for the State-Trait Anxiety Inventory (STAI). Consulting Psychologistics Press, 1983.

[33] Lewis G, Pelosi AJ, Araya R, Dunn G. Measuring psychiatric disorder in the community: a standardized assessment for use by lay interviewers. Psychol Med 1992; 22(2): 465–86. 1615114 10.1017/s0033291700030415

[34] First M, Gibbon M, Spitzer R, Williams J. Structured Clinical Interview for DSM-IV-TR Axis Disorder (Research Version). NewYork State Psychiatric Institute, 2002.

[35] Zammit S, Odd D, Horwood J, Thompson A, Thomas K, Menezes P. Investigating whether adverse prenatal and perinatal events are associated with non-clinical psychotic symptoms at age 12 years in the ALSPAC birth cohort. Psychol Med 2009; 39(9): 1457–67. 19215630 10.1017/S0033291708005126

[36] Raine A, Benishay D. The SPQ-B: a brief screening instrument for schizotypal personality disorder. J Pers Disord 1995; 9: 346–55.

[37] Foa EB, Huppert JD, Leiberg S, Langner R, Kickic R, Hajcak G, The Obsessive-Compulsive Inventory: development and validation of a short version. Psychol Assess 2002; 14(4): 485–96. 12501574

[38] Vinkhuyzen AAE, Eyles DW, Burne THJ, Blanken LME, Kruithof CJ, Verhulst F, Gestational vitamin D deficiency and autism-related traits: the Generation R study. Mol Psychiatry 2018; 23(2): 240–6. 27895322 10.1038/mp.2016.213 PMC5554617

[39] Gustavsson JP. Validity and Stability of Self-Reported Personality Traits. Contributions to the Evaluation of the Karolinska Scales of Personality. Karolinska Institutet, 1997.

[40] Hesse M, Moran P. Screening for personality disorder with the Standardised Assessment of Personality: Abbreviated Scale (SAPAS): further evidence of concurrent validity. BMC Psychiatry 2010; 10: 10. 20109169 10.1186/1471-244X-10-10 PMC2824652

[41] Saunders JB, Aasland OG, Babor TF, de la Fuente JR, Grant M. Development of the Alcohol Use Disorders Identification Test (AUDIT): WHO collaborative project on early detection of persons with harmful alcohol consumption–II. Addiction 1993; 88(6): 791–804. 8329970 10.1111/j.1360-0443.1993.tb02093.x

[42] Pearson RM, Fernyhough C, Bentall R, Evans J, Heron J, Joinson C, Association between maternal depressogenic cognitive style during pregnancy and offspring cognitive style 18 years later. Am J Psychiatry 2013; 170(4): 434–41. 23318526 10.1176/appi.ajp.2012.12050673 PMC3640292

[43] Haeffel GJ, Gibb BE, Metalsky GI, Alloy LB, Abramson LY, Hankin BL, Measuring cognitive vulnerability to depression: development and validation of the Cognitive Style Questionnaire. Clin Psychol Rev 2008; 28: 824–36. 18234405 10.1016/j.cpr.2007.12.001 PMC4090011

[44] Smith B, Dalen J, Wiggins K, Tooley E, Christopher P, Bernard J. The Brief Resilience Scale: assessing the ability to bounce back. Int J Behav Med 2008; 15: 194–200. 18696313 10.1080/10705500802222972

[45] Goldberg LR. A broad-bandwidth, public-domain, personality inventory measuring the lower-level facets of several five-factor models In Personality Psychology in Europe (ed. I Mervielde): 7–28. Tilburg University Press, 1999.

[46] Eysenck SBG, Eysenck HJ, Barrett P. A revised version of the Psychoticism scale. Pers Individ Dif 1985; 6: 21–9.

[47] Howard DM, Adams MJ, Clarke TK, Hafferty JD, Gibson J, Shirali M, Genome-wide meta-analysis of depression identifies 102 independent variants and highlights the importance of the prefrontal brain regions. Nat Neurosci 2019; 22(3): 343–52. 30718901 10.1038/s41593-018-0326-7 PMC6522363

[48] Royston P, White IR. Multiple imputation by chained equations (MICE): implementation in Stata. J Stat Softw 2011; 45(4). Available from: 10.18637/jss.v045.i04

[49] Lewis G, Duffy L, Ades A, Amos R, Araya R, Brabyn S, The clinical effectiveness of sertraline in primary care and the role of depression severity and duration (PANDA): a pragmatic, double-blind, placebo-controlled randomised trial. Lancet Psychiatry 2019; 6(11): 903–14. 31543474 10.1016/S2215-0366(19)30366-9 PMC7029306

[50] Carpenter JK, Andrews LA, Witcraft SM, Powers MB, Smits JAJ, Hofmann SG. Cognitive behavioral therapy for anxiety and related disorders: a meta-analysis of randomized placebo-controlled trials. Depress Anxiety 2018; 35(6): 502–14. 29451967 10.1002/da.22728 PMC5992015

[51] Malhi GS, Mann JJ. Depression. Lancet 2018; 392: 2299–312. 30396512 10.1016/S0140-6736(18)31948-2

[52] Craske MG, Stein MB. Anxiety. Lancet 2016; 388(10063): 3048–59. 27349358 10.1016/S0140-6736(16)30381-6

[53] Wright L, Steptoe A, Fancourt D. How are adversities during COVID-19 affecting mental health? Differential associations for worries and experiences and implications for policy. medRxiv [Preprint] 2020 Available from: https://www.medrxiv.org/content/10.1101/2020.05.14.20101717v2 [cited 9 July 2020].

[54] Druss BG. Addressing the COVID-19 pandemic in populations with serious mental illness. JAMA Psychiatry 2020; 77: 891–2. 32242888 10.1001/jamapsychiatry.2020.0894

[55] Yao H, Chen J-H, Xu Y-F. Patients with mental health disorders in the COVID-19 epidemic. Lancet Psychiatry 2020; 7(4): e21. 32199510 10.1016/S2215-0366(20)30090-0 PMC7269717

[56] Shore JH, Schneck CD, Mishkind MC. Telepsychiatry and the coronavirus disease 2019 pandemic-current and future outcomes of the rapid virtualization of psychiatric care. JAMA Psychiatry [Epub ahead of print] 11 May 2020 Available from: https://jamanetwork.com/journals/jamapsychiatry/fullarticle/10.1001/jamapsychiatry.2020.1643. 10.1001/jamapsychiatry.2020.1643 32391861

[57] Netsi E, Pearson RM, Murray L, Cooper P, Craske MG, Stein A. Association of persistent and severe postnatal depression with child outcomes. JAMA Psychiatry 2018; 75: 547–53. 29387878 10.1001/jamapsychiatry.2017.4363 PMC5885957

[58] Kwong ASF. Examining the longitudinal nature of depressive symptoms in the Avon Longitudinal Study of Parents and Children (ALSPAC). Wellcome Open Res 2019; 4: 126. 31595229 10.12688/wellcomeopenres.15395.1 PMC6764237

