# Supplementary material for: Mental health before and during the COVID-19 pandemic in two longitudinal UK population cohorts
Source: Br J Psychiatry. 2020 Nov 24:1–10. doi: 10.1192/bjp.2020.242 (PMC7844173; doi:10.1192/bjp.2020.242)
Supplement: Supplementary file 1 [file S0007125020002421sup001.docx]

**Mental health before and during COVID-19 in two longitudinal UK population cohorts
- Supplement**

**Supplement methods**

**Supplement figure 1.** Flow diagram for ALSPAC-parents
**Supplement figure 2.** Flow diagram for ALSPAC-young
**Supplement figure 3.** Flow diagram for Generation Scotland

**Supplement table 1.** Sample characteristics for each cohort
**Supplement table 2.** Frequency of missing covariates in ALSPAC-parents
**Supplement table 3.** Frequency of missing covariates in ALSPAC-young
**Supplement table 4.** Frequency of missing covariates in GS
**Supplement table 5.** Proportion above thresholds and mean scores for mental health during COVID-19 in ALSPAC-young
**Supplement table 6.** Proportion above thresholds and mean scores for mental health during COVID-19 in ALSPAC-parents
**Supplement table 7.** Ratio and mean differences between mental health during COVID-10 in ALSPAC-young

**Supplement figure 4.** Mental health during COVID-19 across cohorts **Supplement figure 5.** Mental health during COVID 19 for each mental health condition by ALSPAC and Generation Scotland **Supplement figure 6.** Item level analysis between pre-pandemic and COVID-19 assessments in ALSPAC-young
**Supplement figure 7.** Predicted trajectories of mental health during COVID-19 in ALSPAC-young

**Supplement table 8.** Associations between pre-pandemic risk factors and depression and anxiety using the imputed samples **Supplement table 9.** Associations between pre-pandemic risk factors and depression and anxiety using the complete case samples
**Supplement table 10.** Associations between pre-pandemic risk factors and depression and anxiety, adjusting for educational background using the imputed samples
**Supplement table 11.** Association between pre-pandemic risk factors and depression during COVID-19 with imputation and complete case data at different pre-pandemic timings in ALSPAC-young
**Supplement table 12.** Associations between baseline factors and anxiety symptoms during COVID-19 with imputation and complete case data at different pre-pandemic timings in ALSPAC-young
**Supplement table 13.** Associations between financial problems and depression and anxiety symptoms during COVID-19 with different pre-pandemic timings of financial problems in ALSPAC-young
**Supplement table 14.** Logistic regressions between factors and depression and anxiety using complete case data

**Supplement methods**

**Information about pre-pandemic and COVID-19 factors associated with COVID-19 mental health**

The factors highlighted within this study have been selected based on the existing literature on factors for poorer mental health in and out of pandemics. Where possible we have tried to compare across all cohorts with harmonised assessments, but in some instances, measures were not available or identical. Where measurements differed, we took validated thresholds or standardised scores to compare between cohorts. These are highlighted in Table 1 in the manuscript, with further details on each measure in each cohort given below.

**ALSPAC-parents factors (also known as ALSPAC-G0 or the original parents)**

**Sociodemographic factors**

**Sex:** The sex of the participant was recorded at the time of the COVID-19 questionnaire. Where this was missing, historical data from previous questionnaires and clinics was used to identify the sex of the participant. Sex was coded as 0 - males and 1 - females.
**Age:** The age of the participant was recorded at the time of the COVID-19 questionnaire. Where this was missing, historical data from previous questionnaires and clinics was used to identify the current of the participant. Age at the COVID-19 questionnaire was recoded in years and ranged from 42 years to 81 years (mean: 58.7 years, SD: 4.82).
**Educational background:** Educational background was assessed through questionnaire items assessed at the beginning of recruitment for the study and assessed educational attainment which was split into the following categories indicting higher to lower attainment: 0 - A-Level or higher, 1 – O-Level and 2 - < O-Level. For parsimony, the estimates presented in this analysis compares those in the ‘0 - A-Level or higher’ with those from ‘2 - < O-Level’.
**Income:** Recent monthly household income was assessed at the most recent questionnaire between 2011-2014. Income was split into deciles with 1 indicting lower income (< £899 a month) and 10 indicting higher income (> £4001 a month).
**Neighbourhood deprivation:** Deprivation was measured using indexes of multiple deprivation (IMD) scale from geographically coded data derived in January 2014. IMD was split in quintiles with the 1 indicating lowest deprivation and 5 indicating highest deprivation.
**Financial problems:** Recent financial problems were assessed between 2010-2013 using a questionnaire item which asked “Have you experienced major financial problems in the last year? Responses were: 0 – no, did not happen, 1 - yes, but did not affect me, 2 – yes, mildly affected, 3 – yes, moderately affected and 4 – yes, affected me a lot. We collapsed categories 1 through 4 to indicate financial problems.
**Partner emotional abuse:** Recent emotional abuse was assessed between 2010-2013 using a questionnaire item which asked “Was your partner emotionally cruel to you in the last year? Responses were: 0 – no, did not happen, 1 - yes, but did not affect me, 2 – yes, mildly affected, 3 – yes, moderately affected and 4 – yes, affected me a lot. We collapsed categories 2 through 4 to indicate partner emotional abuse problems.

**Physical health factors**

**Obesity:** Obesity was assessed at the most recent clinic visit between 2011-2015. Obesity was derived from BMI, which was coded as 0 – underweight (BMI ≤ 18.49), 1- healthy range (BMI ≥ 18.5 & ≤ 24.99), 2 – overweight (BMI ≥ 25 & ≤ 29.99) and 3 – obese (BMI ≥ 30). For parsimony, the estimates presented in this analysis compares those in the ‘1- healthy range’ with those from ‘3 – obese’.
**Asthma:** A history or asthma was assessed by a questionnaire item which asked “Have you ever had asthma? between 2002-2004.

**COVID-19 specific factors**

**COVID-19 infection:** A COVID-19 infection was assessed during the COVID-19 questionnaire with the question “Do you think you have or have had COVID-19?” Responses were 0 – no, 1 – yes, but suspected and 2 – yes, confirmed with test”. We collapsed categories 1 and 2 to indicate a COVID-19 infection. Note: In the UK there was a shortage of tests and during the pandemic and individuals were told to self-isolate with symptoms and if these worsened, call the National Health Service helpline or an ambulance. Thus, many did not have access to a diagnostic test.
**Self-isolation:** Self-isolation was assessed during the COVID-19 questionnaire with the question: “Are you currently self-isolating/have you self-isolated?” Responses were coded: 0 – no and 1 – yes.
**Living alone during COVID-19:** Living alone was assessed during the COVID-19 questionnaire with the question: “How many people do you currently live with?” We tallied the responses and then created four groups: 0 – those living alone, 1 – those living with one other person. 2 – those living with 2 to 4 individuals and 3 – those living with more than 5 individuals. For parsimony, the estimates presented in this analysis compares those in the ‘0 - living alone with ‘1 – those living with one other person’. Results from the main analysis were identical when collapsing categories 1 through 3 in sensitivity analysis.
**No access to a garden:** Access to a garden was assessed during the COVID-19 questionnaire with the question: “Do you have access to a shared/private garden?” Responses were coded:0 – yes and 1 – no.
**Health care worker status:** Heath care worker status was assessed during the COVID-19 questionnaire with the question: “Are you a health care worker?” Responses were coded to 0 – no (or yes but in the past) and 1 – yes currently.
**Key worker status:** Key worker status was assessed during the COVID-19 questionnaire with the following question: “Are you a key worker defined by the government?” Responses were coded: 0 – no and 1 – yes.

**Pre-pandemic mental health measures**

**Depressive symptoms:** Pre-pandemic depressive symptoms were measured using the Edinburgh Postnatal Depression Scale or EPDS, [1] between 2011-2013. The EPDS consists of 10 items probing depressive symptoms in the last two weeks with scores ranging between 0-30 and higher scores indicating greater depression. Scores ≥ 12 have been validated against probable major depression. [2]
**Anxiety symptoms:** Pre-pandemic anxiety symptoms were measured using the Spielberger State Trait Anxiety Inventory or STAI, [3] between 1999-2001. We used the trait scale of the STAI, which consists of 20 items with scores ranging between 20-80 with higher scores indicating greater trait anxiety. Scores ≥ 46 have been validated to predict a moderate anxiety disorder. [3]

**Pre-pandemic psychiatric or mental health factors**

**Probable depression:** Probable depression was assessed using questionnaire items between 2001 and 2003, which asked “Have you ever had severe depression?” We coded responses as 0 – no and 1 – yes (yes in the past year or yes, but not recently).
**Probable anxiety:** Probable anxiety was assessed using questionnaire items between 2003 and 2005, which asked “Have you ever had anxiety or nerves?” We coded responses to 0 – no (including yes, but did not consult a doctor) and 1 – yes (and consulted a doctor).
**Disordered eating:** Disordered eating was assessed using questionnaire items between 2001 and 2003, which asked “Have you ever had bulimia?” and “Have you ever had anorexia nervosa?” We coded responses to be 0 – no to both and 1 – yes if either was endorsed.
**Personality disordered traits:** Personality disordered traits were assessed using the Karolinska Scales of Personality (KSP) inventory. [4] between 2000 and 2002. The KSP examines fifteen sub-scales of personality including somatic anxiety, psychic anxiety, muscular tension, psychasthenia, inhibition of aggression, irritability, guilt, socialization, social desirability, monotony avoidance, impulsivity, verbal aggression, indirect aggression, suspicion and detachment. We used methods described previously, [5] to identify individuals who scored in the top quartile of the KSP to define individuals with high personality disorder traits.
**History of alcohol misuse:** A history of alcohol misuse was assessed using the Alcohol Use Disorders Identification Test (AUDIT), [6] between 2011-2013. The AUDIT consists of 10 items with scores ranging between 0-40. Higher scores indicate greater alcohol misuse and we used the threshold of ≥ 8 to indicate hazardous levels of alcohol consumption. [6]
**Current smokers:** Current smokers (tobacco) were assessed using questionnaire items asking “Do you currently smoke?” and if yes “Do you smoke every day?”, assessed between 2011-2013. We coded responses as 0 – not smokers (or those who don’t smoke every day) and 1- yes, smoke every day
**Cognitive styles:** Negative cognitive styles were assessed using six questions about negative cognitive schemas such as: “I avoid saying what I think for fear of being rejected”, “If others knew the real me, they would not like me”, “If other people knew what I am really like, they would think less of me”, “I always expect criticism”, “I don’t like people to really know me” and “My value as a person depends enormously on what others think of me”. These were assessed between 2011 -2013 and derived from previous research. [7] Each question ranged between 0-4 with higher scores representing more negative schemas. We summed up the scores for the six questions resulting in a score between 0-18, with higher scores indicting more negative cognitive styles, in line with previous research. [7] To ease interpretation, we created a standardised score to have a mean of 0 and a standard deviation of 1.
**Depression polygenic risk score:** The depression polygenic risk score (PRS) was created using summary statistics taken from a recent GWAS on major depression. [8] Further details on genotyping and PRS creating are given below.

**ALSPAC-young factors (also known as ALSPAC-G1 or the original index participants)**

**Sociodemographic factors**

**Sex:** The sex of the participant was recorded at the time of the COVID-19 questionnaire. Where this was missing, historical data from previous questionnaires and clinics was used to identify the sex of the participant. Sex was coded as 0 - males and 1 - females.
**Age:** The age of the participant was recorded at the time of the COVID-19 questionnaire. Where this was missing, historical data from previous questionnaires and clinics was used to identify the current of the participant. Age at the COVID-19 questionnaire was recoded in years and ranged from 27 years to 29 years (mean: 27.61 years, SD: 0.54).
**Educational background:** Educational background was assessed via parental educational attainment at enrolment into the study and was split into the following categories indicting higher to lower attainment: 0 - A-Level or higher, 1 – O-Level and 2 - < O-Level. For parsimony, the estimates presented in this analysis compares those in the ‘0 - A-Level or higher’ with those from ‘2 - < O-Level’.
**Income:** Monthly income was assessed at the most recent questionnaire between 2017-2018. Income was split into eight categories with 0 indicating not doing paid work, 1 indicting lower income (£1 - £499 a month) and 7 indicting higher income (£3000 + a month). Results from the main analysis were identical when removing those not doing paid work in sensitivity analysis.
**Neighbourhood deprivation:** Deprivation was measured using indexes of multiple deprivation (IMD) scale from geographically coded data derived in January 2014. IMD was split in quintiles with the 1 indicating lowest deprivation and 5 indicating highest deprivation.
**Financial problems:** Recent financial problems were assessed between 2018-2020 using a questionnaire item which asked “Have you experienced major financial problems in the last year?” Responses were: 0 – no, did not happen, 1 - yes, but did not affect me, 2 – yes, mildly affected, 3 – yes, moderately affected and 4 – yes, affected me a lot. We collapsed categories 1 through 4 to indicate financial problems.
**Parent with young children:** Being a parent with young children was assessed through historical questionnaire items which have asked if the participant was a parent since 2012 (when participants were approximately 19 years old) and continued in subsequent questionnaires.

**Physical health factors**

**Obesity:** Obesity was assessed at the most recent clinic visit between 2015-2017. Obesity was derived from BMI, which was coded as 0 – underweight (BMI ≤ 18.49), 1- healthy range (BMI ≥ 18.5 & ≤ 24.99), 2 – overweight (BMI ≥ 25 & ≤ 29.99) and 3 – obese (BMI ≥ 30). For parsimony, the estimates presented in this analysis compares those in the ‘1- healthy range’ with those from ‘3 – obese’.
**Asthma:** Asthma was assessed by a questionnaire item which asked “Are you currently using any medication for asthma?” between 2014-2015.

**COVID-19 specific factors**

These questions were derived identically to those in ALSPAC-parents.

**Pre-pandemic mental health measures**

**Depressive symptoms:** Pre-pandemic depressive symptoms were measured using the Depressive symptoms in ALSPAC were measured using the Short Mood and Feelings Questionnaire (SMFQ), [9] a 13-item instrument examining depressive mood within the last two weeks. The SMFQ was assessed between 2017-2018 and scores range between 0-26 with higher scores indicting higher depressive symptoms. Scores of ≥11 on the SMFQ have good specificity and sensitivity for probable depression. [10]
**Anxiety symptoms:** Anxiety symptoms were measured using the Generalised Anxiety Disorder Assessment (GAD-7), [11] a 7-item instrument which measures the presence of generalised anxiety disorder symptoms within the last two weeks. The GAD-7 was assessed between 2014-2015 and scores range between 0-21 with higher scores indicting higher anxiety symptoms and scores ≥10 have good specificity and sensitivity for probable generalised anxiety disorder. [12]
**Mental wellbeing:** Mental wellbeing was measured using the Short Warwick-Edinburgh Mental Wellbeing Scale (SWEMWBS), [13] a 7-item instrument which measures positive mental wellbeing within the last two weeks. The SWEMWBS was assessed in 2015-2016 and scores range between 7-35, with higher scores indicating better mental wellbeing. Scores ≤17 on SWEMWBS have been shown to be indicative of probable depression. [14]

**Pre-pandemic psychiatric or mental health factors**

**Probable depression:** Probable depression was assessed using a computerised version of the Clinical Interview Schedule-Revised (CIS-R), [15] which derives a diagnosis of depression according to International Classification of Diseases, 10th Revision criteria. The CIS-R was assessed at two research clinics between 2008-2010 and 2015-2017. We derived probable depression as having a diagnosis at either occasion.
**Probable anxiety:** Probable generalised anxiety disorder (GAD) was assessed using a computerised version of the Clinical Interview Schedule-Revised (CIS-R), [15] which derives a diagnosis of GAD according to International Classification of Diseases, 10th Revision criteria. The CIS-R was assessed at two research clinics between 2008-2010 and 2015-2017. We derived probable GAD as having a diagnosis at either occasion.
**Psychosis like experiences:** Psychosis like experiences were assessed using the Psychosis Like Symptoms Interview (PLIKSi), [16] a structured interview measuring the presence of psychotic like experience such as visual/auditory hallucinations, delusions and thought interference. The PLIKSi is administered by an interviewer and was assessed at two research clinics between 2008-2010 and 2015-2017. We derived a diagnosis of psychosis like experiences if a participant was rated as having suspected or definite psychosis like symptoms at either occasion.
**Disordered eating:** Disordered eating was derived using questionnaire items consistent with DSM-V frequency on the following behaviours: fasting, purging, loss of controlled eating or excessive eating between 2016-2017. We coded responses to be 0 – no behaviours and 1 – at least one behaviour.
**OCD traits:** Obsessive compulsive disorder (OCD) traits were assessed using a modified 6 item version of the short version of the Obsessive-Compulsive Inventory, [17] between 2016-2017. The questions asked were ‘I get upset if objects are not arranged properly’, ‘I feel I have to repeat certain numbers’, ‘I sometimes have to wash or clean myself simply because I feel contaminated or dirty’, ‘I repeatedly check gas and water taps and light switches after turning them off’ and ‘I am upset by unpleasant thoughts that come into my mind against my will’. Responses ranged from 0 – not true, 1 – sometimes true, 2 – often true and 3 – almost always true. We summed these items to create a score, which was then standardised to have a mean of 0 and a standard deviation of 1.
**Autistic traits:** Autistic traits were measured using a modified 18 item version of the Social Responsiveness Scale - 2 (SRS-2), [18] between 2016-2017. We summed the 18 items to create a summary score of all the items and then multiplied that score by 3.6111 to obtain a full scale that matches the 65 items in the original SRS. We then used a validated threshold of ≥ 68 to indicate mild autistic traits as suggested in previous research. [18]
**Personality disordered traits:** Personality disordered traits were assessed using the Standard Assessment of Personality: Abbreviated Scale (SAPAS), [19] between 2015-2017. The SAPAS contains 8 items with yes or no answers pertaining to areas of personality disorder. We used a cut-off of ≥ 5 (top 11%) to indicate high personality disorder traits. Results were identical when using a standardised score of the SAPAS in sensitivity analysis.
**History of alcohol misuse:** A history of alcohol misuse was assessed at two research clinics between 2008-2010 and 2015-2017. The AUDIT, [6] was assessed at the first research clinic, and we used the threshold of ≥ 16 to indicate cases of harmful levels of alcohol misuse (5.44%). At the second research clinic, alcohol misuse was defined through questions consistent with DSM-IV frequency for alcohol abuse and were coded as 0 – no symptoms of base and 1 – yes symptoms of abuse (9.5%).
**Current smokers:** Current smokers (tobacco) were assessed using questionnaire items at a research clinic between 2015-2017 that asked “Do you currently smoke?” and if yes “Do you smoke every day?” We coded responses as 0 – not smokers (or those who don’t smoke every day) and 1- yes, smoke every day. A history of daily smoking (assessed at between 2008-2010 in addition to 2015-2017) showed similar results in sensitivity analyses.
**Cognitive styles:** Negative cognitive styles were assessed using the Cognitive Styles Questionnaire: Short Form (CSQ-SF) [20], between 2008-2010. Briefly, the CSQ-SF presents eight hypothetical events relating to failures in academic, employment, and interpersonal relationships and score is determined by rating whether this failure was due to specific vs global factors (we restricted analysis to the global factor derived in previous research [7]). Scores range between 16 and 71, with higher scores indicating more negative cognitive styles. We created a standardised score to have a mean of 0 and standard deviation of 1.
**Difficulties access mental health information**: Difficulty in accessing mental health information was assessed using a questionnaire item which asked “How easy or difficult is it for you to find information on how to manage mental health problems like stress or depression? ” between 2017-2018. Responses ranged between 0 – very easy, 1 – easy, 2 – difficult and 3 – very difficult. We coded responses as 0 – very easy or easy and 1 – difficult or very difficult.
**Neuroticism:** Neuroticism was assessed using the five-factor model of personality, with questions taken from International Personality Item Pool (IPIP), [21] and was assessed between 2005-2006. Scores from the questions were summed with higher scores indicating higher neuroticism. We created a standardised score to have a mean of 0 and standard deviation of 1.
**Self-harm history:** Self-harm history was assessed using questionnaire items at two research clinics between 2008-2010 and 2015-2017, which both probed the number of self-harm incidents within the last year. We derived self-harm history if participants had self-harmed within the last year at the first occasion and if they had ever self-harmed at the second research clinic. Results were identical in sensitivity analysis when including individuals who had had only recently self-harmed at both occasions.
**Depression polygenic risk score:** The depression polygenic risk score (PRS) was created using summary statistics taken from a recent GWAS on major depression. [8] Further details on genotyping and PRS creating are given below.

**Generation Scotland factors**

**Sociodemographic factors**

**Sex:** The sex of the participant was recorded at the time of the COVID-19 questionnaire. Where this was missing, historical data from previous questionnaires and clinics was used to identify the sex of the participant. Sex was coded as 0 - males and 1 - females.
**Age:** The age of the participant was recorded at the time of the COVID-19 questionnaire. Where this was missing, historical data from previous questionnaires and clinics was used to identify the current of the participant. Age at the COVID-19 questionnaire was recoded in years and ranged from 27 years to 100 years (mean: 59.24 years, SD: 12.03).
**Educational background:** Educational background was assessed at enrolment into the study and was split into the following categories indicting higher to lower attainment: 0 - A-Level or higher, 1 – O-Level and 2 - < O-Level. For parsimony, the estimates presented in this analysis compares those in the ‘0 - A-Level or higher’ with those from ‘2 - < O-Level’.
**Income:** Yearly household income was assessed at enrolment into the study. Income was split into five categories with 0 indicting lower income (less than £10,000) and 4 indicting higher income (more than £70,000).
**Neighbourhood deprivation:** Deprivation was measured using the Scottish indexes of multiple deprivation (SIMD) scale from geographically coded data at the time of enrolment. IMD was split in quintiles with the 1 indicating lowest deprivation and 5 indicating highest deprivation.
**Financial problems:** Recent financial problems were assessed at the STRADL follow up between 2015-2016 using a questionnaire item which asked “Did you have a major financial crisis such as losing the equivalent of three months income, in the last six months? Responses were: 0 – no, did not happen and 1 - yes, did happen.
**Parent with young children:** Being a parent with young children was assessed at the time of the COVID-19 questionnaire. Questions asked “How many children under the age of 12 the participant currently lives with?” We restricted our analysis to only those individuals who lived with another adult or young children (i.e., we excluded those who lived with young children and more than one adult).

**Physical health factors**

**Obesity:** Obesity was assessed at enrolment. Obesity was derived from BMI, which was coded as 0 – underweight (BMI ≤ 18.49), 1- healthy range (BMI ≥ 18.5 & ≤ 24.99), 2 – overweight (BMI ≥ 25 & ≤ 29.99) and 3 – obese (BMI ≥ 30). For parsimony, the estimates presented in this analysis compares those in the ‘1- healthy range’ with those from ‘3 – obese’.
**Asthma:** A history or asthma was assessed by a questionnaire item at the study enrolment which asked “Have you ever had asthma?

**COVID-19 specific factors**

**COVID-19 infection:** A COVID-19 infection was assessed during the COVID-19 questionnaire with the question “Do you think you have or have had COVID-19?” Responses were 0 – no, 1 – yes, but suspected and 2 – yes, confirmed with test”. We collapsed categories 1 and 2 to indicate a COVID-19 infection. Note: In the UK there was a shortage of tests and during the pandemic and individuals were told to self-isolate with symptoms and if these worsened, call the National Health Service helpline or an ambulance. Thus, many did not have access to a diagnostic test.
**Living alone during COVID-19:** Living alone was assessed during the COVID-19 questionnaire with the question: “Do you currently live alone?” Responses were coded:0 – no and 1 – yes.
**No access to a garden:** Access to a garden was assessed during the COVID-19 questionnaire with the question: “Do you have a garden?” Responses were coded:0 – yes and 1 – no.
**Key worker status:** Key worker status was assessed during the COVID-19 questionnaire with the following question: “When COVID-19 restrictions were put in place, were you designated as a key worker?” Responses were coded: 0 – no and 1 – yes.

**Pre-pandemic mental health measures**

**Depressive symptoms and anxiety symptoms:** Baseline depressive symptoms and anxiety symptoms were measured using the General Health Questionnaire – 28 form (GHQ-28), [22] between 2015-2016. The GHQ-28 is a 28-item instrument which monitors somatic symptoms, anxiety symptoms, social dysfunction and depressive symptoms in the past few weeks. We used the Likert method (0-1-2-3) to sum each sub-scale (7 items in each sub-scale). [23] Scores ranged between 0-21 for both the depression and anxiety sub-scales, with higher scores indicating higher symptoms. We created standardised scores for both sub-scales with a mean of 0 and standard deviation of 1.

**Baseline psychiatric or mental health factors**

**Probable depression:** Probable depression was assessed using the Structured Clinical Interview for DSM-IV Disorders (SCID), [24] at study enrolment. The threshold for lifetime prevalence of MDD follows Diagnostic and Statistical Manual of Mental Disorders (DSM) criteria. We derived diagnosis of probable depression if participants met this criterion.
**Psychosis like experiences:** Psychosis like experiences were assessed using the Brief Schizotypal Personality Questionnaire (SPQ-B), [25] assessed at study enrolment. The SPQ-B assesses DSM criteria for schizotypal personality disorder (SPD) such as ideas of reference, odd beliefs, unusual perceptual experiences and suspicious/paranoid ideation. We used the cognitive-perceptual subscale to indicate psychosis like experiences with higher scores indicting greater cognitive-perceptual schizotypal personality. We then created a standardised score to have a mean of 0 and standard deviation of 1.
**History of alcohol misuse:** A history of alcohol misuse was assessed at study enrolment by asking “How many alcoholic units did you consume last week?” and “Was this more, less or about the same compared to average?”. We coded responses as 0 if individuals were regularly consuming less than 14 units and week and 1 if they were regularly consuming more than 14 units a week.
**Current smokers:** Current smokers (tobacco) were assessed using questionnaire items at study enrolment that asked “Have you ever smoked tobacco?” We coded responses as 0 – not smokers (never smoked, yes in the past but stopped) and 1- yes, currently smoke.
**Cognitive styles:** Negative cognitive styles were assessed using the Brief Resilience Scale (BRS), [26] between 2015-2016. The BRS examines the ability to bounce back from stress and we used 6 items that range ‘strongly agree’ to ‘strongly disagree’ on a five-point scale. A total score was calculated as the mean of the six questions. We then reverse coded this score so that higher scores indicated less resilience. We then created a standardised score to have a mean of 0 and standard deviation of 1.
**Neuroticism:** Neuroticism was assessed using the Eysenck Personality Questionnaire – Short Form (EPQ-SF), [27] assessed at study enrolment. The EQP-SF consisted of twelve items related to neuroticism with total scores ranging from 0-12. Higher EPQ-SF scores indicate higher neuroticism. We created a standardised score to have a mean of 0 and standard deviation of 1.
**Self-harm history:** Self-harm history was assessed using record linkage to administrative health data on hospital-treated self-harm (generally defined and including all types of intention self-injury as described in previous research [28]).
**Depression polygenic risk score:** The depression polygenic risk score (PRS) was created using summary statistics taken from a recent GWAS on major depression. [8] Further details on genotyping and PRS creating are given below.

**Genotyping in ALSPAC**

ALSPAC-young were genotyped using the Illumina HumanHap550 quad chip genotyping platforms. The resulting raw genome-wide data were subjected to standard quality control methods. Individuals were excluded on the basis of gender mismatches; minimal or excessive heterozygosity; disproportionate levels of individual missingness (>3%) and insufficient sample replication (IBD < 0.8). Population stratification was assessed by multidimensional scaling analysis and compared with Hapmap II (release 22) European descent (CEU), Han Chinese, Japanese and Yoruba reference populations; all individuals with non-European ancestry were removed. SNPs with a minor allele frequency of < 1%, a call rate of < 95% or evidence for violations of Hardy-Weinberg equilibrium (P < 5E-7) were removed. Cryptic relatedness was measured as proportion of identity by descent (IBD > 0.1). Related subjects that passed all other quality control thresholds were retained during subsequent phasing and imputation. 9,115 subjects and 500,527 SNPs passed these quality control filters.

ALSPAC mothers were genotyped using the Illumina human660W-quad array and genotypes were called with Illumina GenomeStudio. PLINK (v1.07) was used to carry out quality control measures on an initial set of 10,015 subjects and 557,124 directly genotyped SNPs. SNPs were removed if they displayed more than 5% missingness or a Hardy-Weinberg equilibrium P value of less than 1.0e-06. Additionally, SNPs with a minor allele frequency of less than 1% were removed. Samples were excluded if they displayed more than 5% missingness, had indeterminate X chromosome heterozygosity or extreme autosomal heterozygosity. Samples showing evidence of population stratification were identified by multidimensional scaling of genome-wide identity by state pairwise distances using the four HapMap populations as a reference, and then excluded. Cryptic relatedness was assessed using a IBD estimate of more than 0.125 which is expected to correspond to roughly 12.5% alleles shared IBD or a relatedness at the first cousin level. Related subjects that passed all other quality control thresholds were retained during subsequent phasing and imputation. 9,048 subjects and 526,688 SNPs passed these quality control filters.

We combined 477,482 SNP genotypes in common between the sample of mothers and sample of children. We removed SNPs with genotype missingness above 1% due to poor quality (11,396 SNPs removed) and removed a further 321 subjects due to potential ID mismatches. This resulted in a dataset of 17,842 subjects containing 6,305 duos and 465,740 SNPs (112 were removed during liftover and 234 were out of HWE after combination). We estimated haplotypes using ShapeIT (v2.r644) which utilises relatedness during phasing. We obtained a phased version of the 1000 genomes reference panel (Phase 1, Version 3) from the Impute2 reference data repository (phased using ShapeIt v2.r644, haplotype release date Dec 2013). Imputation of the target data was performed using Impute V2.2.2 against the reference panel (all polymorphic SNPs excluding singletons), using all 2186 reference haplotypes (including non-Europeans). This gave 8,237 eligible children and 8,196 eligible mothers with available genotype data after exclusion of related subjects using cryptic relatedness measures described previously.

**Genotyping in Generation Scotland**

Genotyping in GS has been described in detail previously, [29] but briefly, autosomal genotype data were available for all GS:SFHS individuals in the present study (n = 18725). Genotypes were imputed using the Haplotype Reference Consortium reference panel (HRC.r1-1) via the Sanger Imputation Server pipeline (https://imputation.sanger.ac.uk). Prior to imputation, individuals with missingness ≥ 3% were excluded, as were SNPs with a call rate of ≤98%, Hardy Weinberg Equilibrium (HWE) P-value ≤ 1 × 10−6, and a minor allele frequency (MAF) ≤ 1%. Phasing of genotype data was performed using the SHAPEIT2 algorithm utilizing the duoHMM option, which refines phasing by utilizing pedigree information. Imputation was performed using PBWT software. Multi-allelic variants, monomorphic variants and SNPs with an imputation INFO score < 0.8 were removed. Population outliers (more than 6SDs from the mean of the first principal component (PC)) were identified and removed from the sample, as were one from each of 52 monozygotic twin pairs, identified by IBD (preferentially retaining cases), and 7 individuals who matched samples from the Psychiatric Genomics Consortium, identified using genotype checksums. After imputation, individuals with missingness ≥ 2%, and genotype with a call rate of ≤98%, MAF ≤ 0.5% and HWE P-value ≤ 1E-05 were excluded using PLINK version 1.9. Strand ambiguous SNPs with 40% ≤ MAF ≤ 50% were also excluded.

**PRS construction in ALSPAC**

The PRS for depression was created in PRSice2, [30] using summary statistics from a recent genome wide association study (GWAS) of major depression. [8] The PRS was created by weighting the effect sizes of up to 164,451 single nucleotide polymorphisms (SNPs) associated with depression symptoms from the initial GWAS at the *P*-value threshold of 5x10^-2^, shown in previous studies to predict the highest amount of variance. [31]. The PRS was standardised to have a mean of 0 and a standard deviation of 1, thus a higher PRS represents higher liability to depression symptoms. We included SNPs that had a MAF of > 1% and info score > 80%) and excluded SNPs with an R^2^ of >0.1, if they were within 250Kb of each other. We excluded SNPs located in the extended MHC region (chromosome 6 (26-33Mb)).

**PRS construction in Generation Scotland**

The PRS for depression was also created in PRSice2 using the same using summary statistics. The PRS was created by weighting the effect sizes of up to 159,974 single nucleotide polymorphisms (SNPs) associated with depression symptoms from the initial GWAS at the *P*-value threshold of 5x10^-^2. The PRS was also standardised to have a mean of 0 and a standard deviation of 1, thus a higher PRS represents higher liability to depression symptoms. We included SNPs that had a MAF of > 1% and info score > 0.9 and clumped SNPs with R^2^ of >0.1 over a 500kbp sliding window. We excluded SNPs located in the extended MHC region (chromosome 6 (26-33Mb)).

**Additional statistical methods in ALSPAC and GS**

Our main analysis in the manuscript for answering question two examined how pre-pandemic and COVID-19 specific factors were associated with depression and anxiety during COVID-19, adjusting for pre-pandemic depression or anxiety waves (i.e., depression and anxiety at the most recent wave of assessment before COVID-19), and sex, age and when the completed the COVID-19 questionnaire. We included pre-pandemic measures of depression and anxiety as covariates into our regression models. This means that the shared variance between pre-pandemic and COVD-19 depression and anxiety were accounted for. Thus, the remaining variance represents the difference between pre-pandemic and COVID-19 symptoms. The adjusted coefficients represent the extent to which the factors are associated with depression and anxiety in COVID-19, independent of prior mental health.

For this analysis, we treated depression and anxiety as continuous outcomes for two reasons. The first is that we had greater power to detect estimates from linear regressions, in these analyses, compared to logistic regressions. However supplementary analysis using logistic regressions to obtain odds ratios showed similar results (supplement table 14). Secondly, we were interested in the strength of the standardised estimates for depression and anxiety, which could then be compared to clinically relevant effect sizes. [32] Thirdly, there were heterogeneous measures of depression between ALSPAC and GS, so examining continuous standardised estimates for continuous assessments of depression may be a more harmonised approach when comparing results across cohorts and measures.

In the analysis presented in the manuscript for ALSPAC-parents and GS, we used cluster robust standard errors to account for potentially nested data (either by family structure or through responses nested within families [i.e., spousal responses/effects]).

As a further sensitivity check, we adjusted for educational background in all cohorts using the imputed data. This was mainly since individuals with lower educational backgrounds were less likely to complete the COVID-19 questionnaire, and thus our complete case sample may be biased if not adjusting for educational background that could reduce power and result in bias. The sensitivity analysis adjusting for educational background showed identical results to the main analyses presented in the manuscript (supplement table 10).

Finally, we ran sensitivity analysis in ALSPAC-young to examine how pre-pandemic and COVID-19 factors would be associated with depression and anxiety during COVID-19, using different timings for pre-pandemic mental health (or different waves or timings of pre-pandemic mental health). This was because pre-pandemic depressive symptoms and anxiety were measured at different times and therefore the estimates in the regression models could be biased due to different timings of pre-pandemic mental health and proximity to the outcome (COVID-19 mental health). We also explored whether the timing of a pertinent factor (financial problems) may impact on results. Although timings may have differed between cohorts, the assessment of pre-pandemic measures of depression and anxiety provide a ranking for pre-existing mental health vulnerability that must be accounted for when examining mental health in COVID-19. However, these sensitivity results were largely similar to the main analysis presented in the manuscript (supplement tables 11-13), with any substantive changes likely due to changes in sample size or chance.

**Predicted trajectories of mental health during COVID-19 in ALSPAC-young**

Using the wealth of pre-pandemic data in ALSPAC-young, we were able to estimate trajectories of each mental health measure to predict what mental health should have looked like at age 28 (i.e., assuming COVID-19 did not occur), in relation to question one in the main manuscript. We then plotted the observed mental health measure to highlight how mental health in COVID-19 varies from the predicted trajectory.

We used multilevel growth curves to estimate trajectories of depression, anxiety and lower mental wellbeing using StataSE (version 15) and the user written command runmlwin. [33] Further details on estimating multilevel growth curves for depressive symptoms are given in previous research. [34]

Briefly, for depression, we used data from Age 22, Age 24 and Age 26 to estimate quadratic trajectories of depression. We then estimated what the predicted odds of probable depression would be at age 28 (assuming COVID-19 did not occur). We then plotted the observed age 28 (COVID-19) depression odds as a comparison. We restricted analysis to individuals with COVID-19 depression data to ensure the same population was used when estimating trajectories.

For anxiety, we used data from Age 18, Age 22 and Age 24 to estimate quadratic trajectories of probable generalised anxiety disorder (GAD). The GAD-7 was measured at age 22 but it is not possible to make a trajectory with one data point, therefore we supplemented this timepoint with the CISR GAD diagnosis data measured at ages 18 and 24. Therefore these trajectories measure probable anxiety disorder. Similar to the analysis above, we estimated what the odds of probable GAD would be at age 28 (assuming COVID-19 did not occur). We then plotted the observed age 28 (COVID-19) odds of GAD as a comparison. We restricted analysis to individuals with COVID-19 GAD data to ensure the same population was used when estimating trajectories.

For the SWEMWBS, we used data from Age 18 and Age 24 to estimate linear trajectories of mental wellbeing. Linear trajectories were used as it is not possible to examine non-linearity with only two time points. Like the analysis above, we estimated what the predicted odds of lower mental wellbeing scores would be at age 28 (assuming COVID-19 did not occur). We then plotted the observed age 28 (COVID-19) lower mental wellbeing odds as a comparison. We restricted analysis to individuals with COVID-19 mental wellbeing data to ensure the same population was used when estimating trajectories.

In all analysis, we used a random intercept and fixed slope model to estimate trajectories. This is due to lack of variability in outcomes (0 or 1) which make it difficult to accurately estimate random slopes. However, as we were plotting overall population changes (and not individual specific trajectories), this makes little difference on the overall inferences.

**Missing data and Imputation strategy**

The majority (>80%) of participants had more than 50% of complete pre-pandemic data (i.e., all factors and pre-pandemic mental health) with less than 1% only having information on only 1 or 2 pre-pandemic variables. Full information on the proportion of missing pre-pandemic data in each cohort are given in Supplement Tables 2-4.

To address potential bias and loss of power from partial attrition in the cohorts (i.e. many individuals did not have information on all variables), we imputed pre-pandemic depression, anxiety and factors using information that individuals with incomplete data had on earlier mental health or factors, as well as auxiliary variables that were associated with characteristics of those who had partial data. Imputation of missing data was conducted up to the eligible samples in each cohort (i.e., those with complete COVID mental health data). This was done for several reasons. The first is that multiple imputation allows use of all available data, allowing a consistent sample for analysis across different factors (each with a different pattern of partial missing data). This was important as the complete case analysis for individuals with all factors and pre-pandemic and COVID-19 mental health data was small (803 in ALSPAC-parents, 337 in ALSPAC-young and 1174 in GS), however majority of participants were only missing a small % of variables meaning they did provide useful information. Therefore, multiple imputation can increase statistical power by utilising more observations. Secondly, we demonstrated that those with incomplete data were more likely to be from lower education backgrounds. Therefore, the complete case analysis might be biased if we did not adjust for educational background in our models. However, we did not want to present estimates adjusted for education as the primary objective of these results was to highlight vulnerable groups, and not establish causality. Thus, adjusting educational background in the complete case analysis may bias associations between factors and depression during COVID-19, especially if the factor is conditional on educational background (e.g. if the factor is a collider). If education is a confounder for some factors it could result in attenuation and suggest that groups with that factor are not vulnerable. For example, if higher education is associated with reduced likelihood of living alone and increased risk of depression during COVID-19 adjustment for it could attenuate to the null the association between living alone and depression and those living alone would no longer be identified as a vulnerable group who should be monitored.

We used multiple imputation by chained equations to generate 50 imputed datasets, [35] using information from all the factor variables included in our analyses (i.e., MDD, GAD, income, deprivation…) plus additional information from auxiliary variables associated with observed data and missing-ness that would make the missing at random assumption more plausible.

In ALSPAC-parents, the following variables (in addition to the other factors) were included in the multiple imputation model: nine previous assessments of depressive symptoms (EPDS), self-esteem, recent partner physical cruelty, social class during enrolment and family financial problems at enrolment.

In ALSPAC-young, the following variables (in addition to the other factors) were included in the multiple imputation model: nine previous assessments of depressive symptoms (SMFQ), mental wellbeing at 18, generalised anxiety symptoms scores taken from a structure computer interview at age 24, NEET status at age 24 and 26 years, parental social class at birth, family financial problems at birth, parental smoking during pregnancy and the birth order of the participant.

In Generation Scotland, the following variables (in addition to the other factors) were included in the multiple imputation model: social dysfunction, somatic symptoms (both GHQ-28), interpersonal deficits, disorganisation (both SPQ) and extraversion (EPQ).

Predictive mean matching was used for non-normally distributed variables. The parameter estimates for each imputation were combined using Rubin’s rules as applied by the ‘mi estimate’ package in Stata. The missing data patterns are given below in Supplement tables 2-4.

**Further sensitivity analysis in Generation Scotland**

As Generation Scotland is a family-based study, traditional analysis methods may bias the results by failing to account for family structure. [28] Sensitivity analysis in GS was used to quantify this bias by controlling for family structure. Analysis using complete case and imputed data in Generation Scotland were performed in ASReml-R version 4.1. We fitted multilevel models in Generation Scotland to account for family structure. We included the covariates as fixed effects and fit two random effects: an additive genetic effect conditioned on a numerator relationship matrix constructed from a pedigree; and a shared family environment effect based on family ID. For the depression PRS predictor, we used a genomic relationship matrix (GRM) instead of the pedigree matrix to fit the additive genetic effect (note the PRS results are for complete case analysis only, i.e., we did not impute missing PRS). Estimates were similar to the unstructured imputed data, and so those are presented in the manuscript to match the methods and results in ALSPAC. The complete case data is presented below for linear and logistic associations.

**References**

1. Cox, J.L., J.M. Holden, and R. Sagovsky, *Detection of Postnatal Depression: Development of the 10-item Edinburgh Postnatal Depression Scale.* British Journal of Psychiatry, 1987. **150**: p. 782-786.

2. Pearson, R.M., et al., *Maternal depression during pregnancy and the postnatal period: risks and possible mechanisms for offspring depression at age 18 years.* JAMA Psychiatry, 2013. **70**(12): p. 1312-9.

3. Spielberger, C.D., et al., *Manual for the State-Trait Anxiety Inventory STAI.* Consulting Psychologistics Press, 1983.

4. Gustavsson, J.P., *Validity and Stability of Self-reported Personality Traits. Contributions to the Evaluation of the Karolinska Scales of Personality.* 1997: Stockholm: Karolinska Institutet.

5. Pearson, R.M., et al., *Impact of dysfunctional maternal personality traits on risk of offspring depression, anxiety and self-harm at age 18 years: a population-based longitudinal study.* Psychological Medicine, 2017: p. 1-11.

6. Saunders, J.B., et al., *Development of the Alcohol Use Disorders Identification Test (AUDIT): WHO Collaborative Project on Early Detection of Persons With Harmful Alcohol Consumption--II.* Addiction, 1993. **88**(6): p. 791-804.

7. Pearson, R.M., et al., *Association Between Maternal Depressogenic Cognitive Style During Pregnancy and Offspring Cognitive Style 18 Years Later.* Am J Psychiatry, 2013. **170**(4): p. 434-441.

8. Howard, D.M., et al., *Genome-wide meta-analysis of depression identifies 102 independent variants and highlights the importance of the prefrontal brain regions.* Nat Neurosci, 2019. **22**(3): p. 343-352.

9. Angold, A., et al., *Development of a short questionnaire for use in epidemiological studies of depression in children and adolescents.* International Journal of Methods in Psychiatric Research, 1995. **5**(4): p. 237-249.

10. Turner, N., et al., *Validity of the Short Mood and Feelings Questionnaire in Late Adolescence.* Psychological Assessment, 2014.

11. Spitzer, R.L., et al., *A Brief Measure for Assessing Generalized Anxiety Disorder.* Arch Intern Med, 2006. **166**: p. 1092-1097.

12. Kroenke, K., et al., *Anxiety disorders in primary care prevalence, impairment, comorbidity, and detection.* Ann Intern Medicine, 2007. **146**: p. 317-325.

13. Stewart-Brown, S., et al., *Internal construct validity of the Warwick-Edinburgh Mental Well-being Scale (WEMWBS): a Rasch analysis using data from the Scottish Health Education Population Survey.* Health Qual Life Outcomes, 2009. **7**: p. 15.

14. Warwick Univeristy. *Collect, score, analyse and interpret WEMWBS*. 2020; Available from: <https://warwick.ac.uk/fac/sci/med/research/platform/wemwbs/using/howto/>.

15. Lewis, G., et al., *Measuring psychiatric disorder in the community: a standardized assessment for use by lay interviewers.* Psychological Medicine, 1992. **22**(02).

16. Zammit, S., et al., *Investigating whether adverse prenatal and perinatal events are associated with non-clinical psychotic symptoms at age 12 years in the ALSPAC birth cohort.* Psychol Med, 2009. **39**(9): p. 1457-67.

17. Foa, E.B., et al., *The Obsessive-Compulsive Inventory: Development and validation of a short version.* Psychological Assessment, 2002. **14**(4): p. 485–496.

18. Vinkhuyzen, A.A.E., et al., *Gestational vitamin D deficiency and autism-related traits: the Generation R Study.* Mol Psychiatry, 2018. **23**(2): p. 240-246.

19. Hesse, M. and P. Moran, *Screening for personality disorder with the Standardised Assessment of Personality: Abbreviated Scale (SAPAS): further evidence of concurrent validity.* BMC Psychiatry, 2010. **10**: p. 10.

20. Haeffel, G.J., et al., *Measuring cognitive vulnerability to depression: development and validation of the Cognitive Style Questionnaire.* Clin Psychol Rev, 2008. **28**: p. 824-836.

21. Goldberg, L.R., *A broad-bandwidth, public-domain, personality inventory measuring the lower-level facets of several five-factor models*, in *Personality psychology in Europe*, I. Mervielde, et al., Editors. 1999, Tilburg University Press: Tilburg, The Netherlands. p. 7-28.

22. Goldberg, D., *General Health Questionnaire*. 1978, Windsor: NFER Publishing Company.

23. Navrady, L.B., et al., *Cohort Profile: Stratifying Resilience and Depression Longitudinally (STRADL): a questionnaire follow-up of Generation Scotland: Scottish Family Health Study (GS:SFHS).* Int J Epidemiol, 2018. **47**(1): p. 13-14g.

24. First, M., et al., *Structured Clinical Interview for DSM-IV-TR Axis Disorder (Research Version).* 2002, New York, NY: NewYork State Psychiatric Institute.

25. Raine, A. and D. Benishay, *The SPQ-B: a brief screening instrument for schizotypal personality disorder. .* J Pers Disord, 1995. **9**: p. 346-55.

26. Smith, B., et al., *The Brief Resilience Scale: Assessing the Ability to Bounce Back.* International Journal of Behavioral Medicine, 2008. **15**: p. 194-200.

27. Eysenck, S.B.G., H.J. Eysenck, and P. Barrett, *A revised version of the psychotocism scale.* Pers Individ Dif, 1985. **6**: p. 21-29.

28. Hafferty, J.D., et al., *The role of neuroticism in self-harm and suicidal ideation: results from two UK population-based cohorts.* Soc Psychiatry Psychiatr Epidemiol, 2019. **54**(12): p. 1505-1518.

29. Hall, L.S., et al., *Genome-wide meta-analyses of stratified depression in Generation Scotland and UK Biobank.* Transl Psychiatry, 2018. **8**(1): p. 9.

30. Euesden, J., C.M. Lewis, and P.F. O'Reilly, *PRSice: Polygenic Risk Score software.* Bioinformatics, 2015. **31**(9): p. 1466-8.

31. Wray, N.R., et al., *Genome-wide association analyses identify 44 risk variants and refine the genetic architecture of major depression.* Nat Genet, 2018. **50**(5): p. 668-681.

32. Leckie, G. and C. Charlton, *runmlwin: A Program to Run the MLwiN Multilvel Modeling Software from within Stata.* Journal of Statistical Software, 2013. **52**(11).

33. Kwong, A.S.F., et al., *Identifying Critical Points of Trajectories of Depressive Symptoms from Childhood to Young Adulthood.* J Youth Adolesc, 2019. **48**: p. 815-827.

34. Croft, J., et al., *Association of Trauma Type, Age of Exposure, and Frequency in Childhood and Adolescence With Psychotic Experiences in Early Adulthood.* JAMA Psychiatry, 2018.

**Supplement figure 1.** Flow diagram for ALSPAC-parents. There were 3579 individuals that had at least one assessment of the SMFQ, GAD-7 or SWEMWBS during COVID-19. *13761 mothers were originally recruited with their partners. **Supplement figure 2.** Flow diagram for ALSPAC-young. There were 2828 individuals that had at least one assessment of the SMFQ, GAD-7 or SWEMWBS during COVID-19. **Supplement figure 3.** Flow diagram for Generation Scotland. There were 4208 individuals that had at least one assessment of the PHQ-9, GAD-7 or SWEMWBS during COVID-19.

Completed ALSPAC COVID-19 questionnaire (n = 3720)

Completed SMFQ (n = 3578)

Excluded or did not complete
COVID-19 questionnaire (n = 23802)

ALSPAC Parents originally enrolled in
ALSPAC (n = 27522)*

Completed GAD-7 (n = 3577)

Completed SWEMWBS (n = 3578)

Missing SMFQ

(n = 142)

Missing GAD-7

(n = 143)

Missing SWEMWBS

(n = 142)

Completed ALSPAC COVID-19 questionnaire (n = 2973)

Completed SMFQ (n = 2812)

Excluded or did not complete
COVID-19 questionnaire (n = 11,928)

Children enrolled in ALSPAC with further recruitment (n = 14901)

Completed GAD-7 (n = 2850)

Completed SWEMWBS (n = 2832)

Missing SMFQ

(n = 161)

Missing GAD-7

(n = 123)

Missing SWEMWBS

(n = 141)

Completed ALSPAC COVID-19 questionnaire (n = 4233)

Completed PHQ-9 (n = 4205)

Excluded or did not complete
COVID-19 questionnaire (n = 19851)

Participants originally enrolled in Generation Scotland (n = 24084)

Completed GAD-7 (n = 4186)

Completed SWEMWBS (n = 4205)

Missing PHQ-9

(n = 28)

Missing GAD-7

(n = 47)

Missing SWEMWBS

(n = 28)

|  | **ALSPAC-parents** |  | **ALSPAC-young** |  | **Gen Scot** |  |
| --- | --- | --- | --- | --- | --- | --- |
|  | **Most recent assessment and COVID-19 data** | **Only most recent assessment** | **Most recent assessment and COVID-19 data** | **Only most recent assessment** | **Most recent assessment and COVID-19 data** | **Only most recent assessment** |
| **Sex** |  |  |  |  |  |  |
| Male | 758 (26.34%) | 1178 (32.91%) | 673 (27.35%) | 814 (42.20%) | 1106 (35.19%) | 1943 (39.56%) |
| Female | 2120 (73.66%) | 2401 (67.09%) | 1788 (72.65%) | 1115 (57.80%) | 2037 (64.81%) | 2969 (60.44%) |
| **Education background** |  |  |  |  |  |  |
| A level + | 1626 (59.85%) | 1619 (49.27%) | 1126 (50.52%) | 741 (43.49%) | 2275 (76.70%) | 2832 (63.77%) |
| O level | 794 (29.22%) | 1014 (30.86%) | 743 (33.33%) | 577 (33.86%) | 290 (9.78%) | 465 (10.47%) |
| < O level | 297 (10.93%) | 653 (19.87%) | 360 (16.15%) | 386 (22.65%) | 401 (13.52%) | 1144 (25.76%) |
| **MDD history** |  |  |  |  |  |  |
| No | 2418 (92.22%) | 2684 (92.23%) | 1867 (85.84%) | 1180 (86.83%) | 2452 (86.04%) | 3751 (85.89%) |
| Yes | 204 (7.78% | 226 (7.77%) | 308 (14.16%) | 179 (13.17%) | 398 (13.96%) | 616 (14.11%) |
| **DS history** |  |  |  |  |  |  |
| No | 2436 (85.90%) | 3055 (84.37%) | 1703 (76.75%) | 1339 (74.31%) | No threshold assessed in GHQ | |
| Yes | 400 (14.10%) | 566 (15.63%) | 516 (23.25%) | 463 (25.69%) |  |  |
| **AS history** |  |  |  |  |  |  |
| No | 2111 (84.81%) | 2322 (85.30%) | 1703 (76.75%) | 1339 (74.31%) | No threshold assessed in GHQ | |
| Yes | 378 (15.19%) | 400 (14.70%) | 516 (23.25%) | 463 (25.69%) |  |  |

**Supplement table 1.** Sample characteristics for each cohort by sex, education background, history of depression (MDD), depressive symptoms (DS) history at the most recent baseline, anxiety symptoms (AS) history at the most recent baseline (see Supplement Methods for most measures and most recent assessment for each cohort).Data are n (%). Numbers vary due to missing data. % indicate column percentages. MDD: Major depressive disorder. DS: Depressive Symptoms. AS: Anxiety symptoms.

| **Covariates missing** | **Frequency** | **Percentage (%)** | **Cumulative %** |
| --- | --- | --- | --- |
| **0** | 803 | 22.44 | 22.44 |
| **1** | 724 | 20.23 | 42.67 |
| **2** | 497 | 13.89 | 56.55 |
| **3** | 304 | 8.49 | 65.05 |
| **4** | 182 | 5.09 | 70.13 |
| **5** | 102 | 2.85 | 72.98 |
| **6** | 76 | 2.12 | 75.1 |
| **7** | 54 | 1.51 | 76.61 |
| **8** | 74 | 2.07 | 78.68 |
| **9** | 99 | 2.77 | 81.45 |
| **10** | 155 | 4.33 | 85.78 |
| **11** | 101 | 2.82 | 88.6 |
| **12** | 83 | 2.32 | 90.92 |
| **13** | 60 | 1.68 | 92.6 |
| **14** | 42 | 1.17 | 93.77 |
| **15** | 34 | 0.95 | 94.72 |
| **16** | 39 | 1.09 | 95.81 |
| **17** | 27 | 0.75 | 96.56 |
| **18** | 21 | 0.59 | 97.15 |
| **19** | 33 | 0.92 | 98.07 |
| **20** | 19 | 0.53 | 98.6 |
| **21** | 12 | 0.34 | 98.94 |
| **22** | 8 | 0.22 | 99.16 |
| **23** | 10 | 0.28 | 99.44 |
| **24** | 7 | 0.2 | 99.64 |
| **25** | 4 | 0.11 | 99.75 |
| **26** | 6 | 0.17 | 99.92 |
| **27** | 2 | 0.06 | 99.97 |
| **28** | 1 | 0.03 | 100 |
| **Total** | **3,579** | **100** |  |

**Supplement table 2.** Frequency of missing covariates (factors and pre-pandemic assessments) in ALSPAC-parents for individuals with at least one mental health outcome during COVID-19 (n=3579). The additional pre-pandemic factors are described in the imputation strategy.

| **Covariates missing** | **Frequency** | **Percentage (%)** | **Cumulative %** |
| --- | --- | --- | --- |
| **0** | 334 | 11.63 | 11.63 |
| **1** | 308 | 10.72 | 22.35 |
| **2** | 250 | 8.7 | 31.06 |
| **3** | 203 | 7.07 | 38.13 |
| **4** | 188 | 6.55 | 44.67 |
| **5** | 179 | 6.23 | 50.91 |
| **6** | 141 | 4.91 | 55.81 |
| **7** | 117 | 4.07 | 59.89 |
| **8** | 111 | 3.86 | 63.75 |
| **9** | 101 | 3.52 | 67.27 |
| **10** | 94 | 3.27 | 70.54 |
| **11** | 76 | 2.65 | 73.19 |
| **12** | 69 | 2.4 | 75.59 |
| **13** | 88 | 3.06 | 78.66 |
| **14** | 78 | 2.72 | 81.37 |
| **15** | 70 | 2.44 | 83.81 |
| **16** | 64 | 2.23 | 86.04 |
| **17** | 55 | 1.92 | 87.95 |
| **18** | 40 | 1.39 | 89.35 |
| **19** | 41 | 1.43 | 90.77 |
| **20** | 36 | 1.25 | 92.03 |
| **21** | 29 | 1.01 | 93.04 |
| **22** | 30 | 1.04 | 94.08 |
| **23** | 26 | 0.91 | 94.99 |
| **24** | 12 | 0.42 | 95.4 |
| **25** | 17 | 0.59 | 96 |
| **26** | 25 | 0.87 | 96.87 |
| **27** | 17 | 0.59 | 97.46 |
| **28** | 19 | 0.66 | 98.12 |
| **29** | 13 | 0.45 | 98.57 |
| **30** | 24 | 0.84 | 99.41 |
| **31** | 10 | 0.35 | 99.76 |
| **32** | 6 | 0.21 | 99.97 |
| **33** | 1 | 0.03 | 100 |
| **Total** | **2,872** | **100** |  |

**Supplement table 3.** Frequency of missing covariates (factors and pre-pandemic measures) in ALSPAC-young for individuals with at least one mental health outcome during COVID-19 (n=2872). The additional pre-pandemic factors are described in the imputation strategy.

| **Covariates missing** | **Frequency** | **Percentage (%)** | **Cumulative %** |
| --- | --- | --- | --- |
| **0** | 1,174 | 27.9 | 27.9 |
| **1** | 1,159 | 27.54 | 55.44 |
| **2** | 450 | 10.69 | 66.14 |
| **3** | 125 | 2.97 | 69.11 |
| **4** | 415 | 9.86 | 78.97 |
| **5** | 416 | 9.89 | 88.85 |
| **6** | 200 | 4.75 | 93.61 |
| **7** | 112 | 2.66 | 96.27 |
| **8** | 80 | 1.9 | 98.17 |
| **9** | 18 | 0.43 | 98.6 |
| **10** | 21 | 0.5 | 99.1 |
| **11** | 17 | 0.4 | 99.5 |
| **12** | 17 | 0.4 | 99.9 |
| **13** | 2 | 0.05 | 99.95 |
| **14** | 2 | 0.05 | 100 |
| **Total** | **4208** | **100** |  |

**Supplement table 4.** Frequency of missing covariates (factors and pre-pandemic measures) in GS for individuals with at least one mental health outcome during COVID-19 (n=4208). The additional pre-pandemic factors are described in the imputation strategy.

**Supplement figure 4.** Mental health during COVID-19 in ALSPAC-parents, ALSPAC-young and Generation Scotland (GS). Figure shows probable depression, probable generalised anxiety disorder (GAD) and lower wellbeing by each cohort.

**Supplement figure 5.** Mental health during COVID-19 in ALSPAC and Generation Scotland (GS). Supplement figure 5A (top left) shows probable depression by age groups assessed using the SMFQ in ALSPAC and PHQ-9 in GS. Supplement figure 5B (top right) shows probable GAD by age groups assessed by the GAD-7. Supplement figure 5C (bottom left) shows lower wellbeing by age groups assessed by the SWEMWBS. Note, ALSPAC-young (n=2812) were categorised as 18-40, even though the max age of this cohort is 29 years. Age in ALSPAC-parents was split by the following: Age 40-49 (n=89), Age 50-59 (n=2105), Age 60-69 (n=1455) and Age 70+ (n=71). In GS, Age was split by the following: Age 18-40 (n=356), Age 40-49 (n=534), Age 50-59 (n=964), Age 60-69 (n=1526) and Age 70+ (n=853).

|  | **Thresholds** |  |  |  | **Mean Scores** |  |  |  |
| --- | --- | --- | --- | --- | --- | --- | --- | --- |
|  | **Raw*** |  | **Restricted**** |  | **Raw*** |  | **Restricted **** |  |
|  | **% (95% CIs)** | **n** | **% (95% CIs)** | **n** | **Mean (95% CIs)** | **n** | **Mean (95% CIs)** | **n** |
| **COVID-19** |  |  |  |  |  |  |  |  |
| SMFQ (Age 28) | 18.14 (16.76, 19.61) | 2812 | 17.71 (16.18, 19.36) | 2219 | 6.25 (6.04, 6.45) | 2812 | 6.17 (5.94, 6.40) | 2219 |
| GAD-7 (Age 28) | 24.35 (22.81, 25.96) | 2850 | 22.14 (20.29, 24.11) | 1811 | 6.22 (6.02, 6.41) | 2850 | 5.91 (5.69, 6.17) | 1811 |
| SWEMWBS (Age 28) | 13.27 (12.07, 14.56) | 2872 | 13.13 (11.79, 14.60) | 2231 | 22.06 (21.90, 22.22) | 2872 | 22.15 (21.97, 22.33) | 2231 |
| **Recent baseline** |  |  |  |  |  |  |  |  |
| SMFQ (Age 26) | 24.35 (23.04, 25.70) | 4021 | 23.25 (21.54, 25.06) | 2219 | 6.89 (6.69, 7.09) | 4021 | 6.78 (6.52, 7.04) | 2219 |
| GAD-7 (Age 22) | 12.97 (11.87, 14.15) | 3339 | 12.26 (10.83, 13.85) | 1811 | 4.59 (4.44, 4.74) | 3339 | 4.57 (4.36, 4.77) | 1811 |
| SWEMWBS (Age 24) | 7.59 (6.82, 8.43) | 4166 | 7.40 (6.38, 8.56) | 2231 | 24.49 (24.36, 24.63) | 4166 | 24.60 (24.42, 24.79) | 2231 |
| **Additional times** |  |  |  |  |  |  |  |  |
| SMFQ (Age 24) | 24.71 (23.40, 26.07) | 4022 | 23.93 (22.16, 25.79) | 2123 | 7.04 (6.85, 7.23) | 4022 | 6.89 (6.64, 7.15) | 2123 |
| SMFQ (Age 22) | 18.07 (16.79, 19.42) | 3304 | 17.30 (15.60, 19.13) | 1769 | 5.70 (5.51, 5.89) | 3304 | 5.69 (5.44, 5.94) | 1769 |
| SMFQ (Age 18) | 21.65 (20.47, 22.87) | 4495 | 20.17 (18.43, 22.02) | 1919 | 6.58 (6.43, 6.74) | 4495 | 6.53 (6.29, 6.76) | 1919 |
| GAD (Age 24) | 9.75 (8.87, 10.72) | 3957 | 10.32 (9.10, 11.68) | 2142 | - | - | - | - |
| GAD (Age 18) | 5.74 (5.11, 6.46) | 4561 | 6.54 (5.51, 7.75) | 1895 | - | - | - | - |
| SWEMWBS (Age 18) | 7.43 (6.70, 8.23) | 4495 | 7.39 (6.31, 8.64) | 1962 | 23.66 (23.53, 23.79) | 4495 | 23.71 (23.52, 23.91) | 1962 |

**Supplement table 5.** Proportion above thresholds and mean scores for mental health outcomes at COVID-19 questionnaire and at the varying pre-pandemic assessments (waves) in ALSPAC-young. *Raw refers to the proportion/mean at that time point only (i.e., not restricted or adjusted for any other time point). **Restricted refers to the proportion/mean at that time point restricted to those who have both the outcome measure and the pre-pandemic measure (i.e., to ensure the exact same population is being assessed).

|  | **Thresholds** |  |  |  | **Mean Scores** |  |  |  |
| --- | --- | --- | --- | --- | --- | --- | --- | --- |
|  | **Raw*** |  | **Restricted**** |  | **Raw*** |  | **Restricted **** |  |
|  | **% (95% CIs)** | **n** | **% (95% CIs)** | **n** | **Mean (95% CIs)** | **n** | **Mean (95% CIs)** | **n** |
| **COVID-19** |  |  |  |  |  |  |  |  |
| SMFQ | 4.33 (3.71, 5.05) | 3578 | 3.91 (3.25, 4.69) | 2789 | 2.98 (2.86, 3.01) | 3578 | 2.86 (2.72, 2.99) | 2789 |
| GAD-7 | 9.14 (8.24, 10.1) | 3577 | 9.57 (8.57, 10.66) | 3052 | 3.44 (3.31, 3.58) | 3577 | 3.47 (3.33, 3.62) | 3052 |
| SWEMWBS | 5.89 (5.17, 6.72) | 3578 | - | - | 24.07 (23.93, 24.22) | 3578 | - | - |
| **Recent baseline** |  |  |  |  |  |  |  |  |
| EPDS (2011-2013) | 14.96 (14.11, 15.85) | 6457 | 14.31 (13.05, 15.66) | 2789 | 6.54 (6.40, 6.67) | 6457 | 6.36 (6.17, 6.56) | 2789 |
| STAI (1999-2001) | 15.61 (14.96, 16.28) | 11580 | 15.37 (14.13, 16.69) | 3052 | 34.98 (34.79, 35.17) | 11580 | 35.03 (34.66, 35.39) | 3052 |

**Supplement table 6.** Proportion above thresholds and mean scores for mental health outcomes at COVID-19 questionnaire and at the varying pre-pandemic assessments (waves) in ALSPAC-parents. Note: although all the mental health measures have validated thresholds, as they are different measures, they are limited in how much they can be compared between recent baseline and during COVID-19. *Raw refers to the proportion/mean at that time point only (i.e., not restricted or adjusted for any other time point). **Restricted refers to the proportion/mean at that time point restricted to those who have both the outcome measure and the baseline measure (i.e., to ensure the exact same population is being assessed).

|  | **Thresholds** |  |  | **Mean Scores** |  |  |
| --- | --- | --- | --- | --- | --- | --- |
|  | **% Difference  (95% CIs)** | **Standardised Difference (95% CIs)** | **P-Value** | **Mean Difference (95% CIs)** | **Standardised Difference (95% CIs)** | **P-Value** |
| SMFQ (n=2219)  COVID-19 (Age 28) Vs Age 26 | -5.54 (-3.68, -7.40) | -0.12 (-0.08, -0.17) | 6.18 x 10^-09^ | -0.60 (-0.37, -0.84) | 0.11 (0.06, 0.15) | 4.84 x 10^-07^ |
| GAD-7 (n=1811)  COVID-19 (Age 28) Vs Age 22 | 9.88 (7.77, 12.04) | 0.21 (0.17, 0.26) | 5.53 x 10^-19^ | 1.36 (1.61, 1.12) | 0.26 (0.21, 0.30) | 7.77 x 10^-27^ |
| SWEMWBS (n=2231)  COVID-19 (Age 28) Vs Age 24 | 5.74 (4.17, 7.30) | 0.15 (0.11, 0.19) | 8.71 x 10^-13^ | 2.45 (2.25, 2.65) | 0.51 (0.47, 0.55) | 3.71 x 10^-115^ |

**Supplement table 7.** Absolute % (difference in proportions) and mean differences between mental health outcomes at most recent pre-pandemic assessment and during COVID-19 in ALSPAC-young.

 ******Supplement figure 7.** Predicted trajectories of mental health in ALSPAC-young with predicted and observed values at Age 28.

**Supplement figure 6.** Item level changes in mental health between the most recent pre-pandemic assessment and COVID-19 in ALSPAC-young. Supplement figure 6A (top left) shows how items of the SMFQ (depression) vary from the most recent pre-pandemic assessment (Age 26) to COVID-19. Supplement figure 6B (top right) shows how items of the GAD-7 (anxiety) vary from the most recent pre-pandemic assessment (Age 22) to COVID-19. Supplement figure 6C (bottom left) shows high items from the SWEMWBS (mental wellbeing) vary from the most recent pre-pandemic assessment (Age 24) to COVID-19.

|  | **Depression standardised estimates - *β*_STD_ (95% CIs), *P*** | | | **Anxiety standardised estimates - *β*_STD_ (95% CIs), *P*** | | |
| --- | --- | --- | --- | --- | --- | --- |
|  | **ALSPAC-parents**  **(n=3579)** | **ALSPAC-young**  **(n=2872)** | **Gen Scot**  **(n=4208)** | **ALSPAC-parents**  **(n=3579)** | **ALSPAC-young**  **(n=2872)** | **Gen Scot**  **(n=4208)** |
| **Sociodemographic factors** |  |  |  |  |  |  |
| Sex (female)  (76% / 72% / 64%)* | 0.28 (0.21, 0.34) *P* = 1.46 x 10^-19^ | 0.22 (0.15, 0.29) *P* = 1.20 x 10^-9^ | 0.27 (0.22, 0.32)  *P* = 4.07 x 10^-22^ | 0.26 (0.20, 0.32) *P* = 7.87 x 10^-17^ | 0.40 (0.33, 0.48) *P* = 3.57 x 10^-23^ | 0.18 (0.12, 0.23)  *P* = 6.63 x 10^-10^ |
| Age (older ages)  (scale variable)** | -0.02 (-0.03, -0.01) *P* = 2.08 x 10^-8^ | -0.01 (-0.07, 0.04) *P* = 0.643 | -0.02 (-0.02, -0.02) *P* = 4.57 x 10^-46^ | -0.02 (-0.03, -0.02) *P* = 4.82 x 10^-12^ | 0.00 (-0.06, 0.07) *P* = 0.945 | -0.01 (-0.02, -0.01) *P* = 1.25 x 10^-30^ |
| Lower educational background  (11%/ 16% / 13%) | 0.23 (0.12, 0.35) *P* = 0.00008 | 0.05 (-0.04, 0.15) *P* = 0.276 | 0.15 (0.06, 0.24) *P* = 0.001 | 0.14 (0.03, 0.25) *P* = 0.016 | 0.16 (0.06, 0.26) *P* = 0.002 | 0.09 (0.00, 0.18) *P* = 0.061 |
| Higher income  (scale variable) | -0.04 (-0.06, -0.03) *P* = 6.22 x 10^-8^ | -0.01 (-0.04, 0.01) *P* = 0.328 | -0.10 (-0.13, -0.08)  *P* = 5.14 x 10^-16^ | -0.01 (-0.03, 0.00) *P* = 0.052 | -0.04 (-0.07, -0.01) *P* = 0.008 | -0.07 (-0.09, -0.04)  *P* = 5.46 x 10^-7^ |
| Worse deprivation status (scale variable) | 0.07 (0.04, 0.10) *P* = 0.00002 | 0.01 (-0.01, 0.04) *P* = 0.319 | 0.06 (0.04, 0.09)  *P* = 7.33 x 10^-7^ | 0.05 (0.02, 0.08) *P* = 0.001 | 0.04 (0.01, 0.07) *P* = 0.005 | 0.04 (0.02, 0.06)  *P* = 0.001 |
| Financial problems  (11% / 10% / 4%) | 0.29 (0.15, 0.43) *P* = 0.00005 | 0.14 (0.03, 0.26) *P* = 0.015 | 0.38 (0.18, 0.58)  *P* = 0.0002 | 0.20 (0.07, 0.32) *P* = 0.011 | 0.24 (0.12, 0.36) *P* = 0.0002 | 0.20 (0.00, 0.39)  *P* = 0.049 |
| Partner emotional abuse  (8% / Na / Na) | 0.36 (0.18, 0.53) *P* = 0.00005 | Not assessed | Not assessed | 0.29 (0.13, 0.46) *P* = 0.001 | Not assessed | Not assessed |
| Parent with young children  (Na / 11% / 11%) | Not assessed | 0.03 (-0.07, 0.13) *P* = 0.570 | -0.03 (-0.14, 0.08)  *P* = 0.617 | Not assessed | 0.19 (0.08, 0.30) *P* = 0.001 | 0.05 (-0.05, 0.16)  *P* = 0.353 |
| **Physical health factors** |  |  |  |  |  |  |
| Obesity  (18 % / 14% / 20%) | 0.22 (0.12, 0.32) *P* = 0.00001 | 0.18 (0.06, 0.31) *P* = 0.004 | 0.32 (0.24, 0.40)  *P* = 1.09 x 10^-14^ | 0.14 (0.04, 0.25) *P* = 0.005 | 0.15 (0.03, 0.27) *P* = 0.012 | 0.10 (0.02, 0.18)  *P* = 0.010 |
| Asthma  (16% / 10% / 10%) | 0.07 (-0.02, 0.16) *P* = 0.127 | 0.08 (-0.04, 0.20) *P* = 0.196 | 0.18 (0.07, 0.28)  *P* = 0.001 | 0.07 (-0.02, 0.16) *P* = 0.14 | 0.21 (0.07, 0.35) *P* = 0.003 | 0.12 (0.02, 0.22)  *P* = 0.016 |
| **COVID-19 specific factors** |  |  |  |  |  |  |
| COVID-19 infection  (12% /16% / 8%) | 0.18 (0.07, 0.28) *P* = 0.001 | 0.09 (0.00, 0.17) *P* = 0.045 | 0.17 (0.05, 0.29)  *P* = 0.004 | 0.16 (0.06, 0.27) *P* = 0.003 | 0.08 (-0.02, 0.17) *P* = 0.112 | 0.10 (-0.02, 0.21)  *P* = 0.101 |
| Self-isolation  (19% /25% / Na) | 0.20 (0.11, 0.29) *P* = 6.64 x 10^-6^ | 0.15 (0.08, 0.22) *P* = 0.00004 | Not assessed | 0.13 (0.04, 0.27) *P* = 0.003 | 0.17 (0.09, 0.25) *P* = 0.00003 | Not assessed |
| Living alone  (8% / 6% / 16%) | 0.45 (0.30, 0.59) *P* = 4.80 x 10^-9^ | 0.20 (0.06, 0.34) *P* = 0.005 | 0.19 (0.11, 0.27)  *P* = 4.47 x 10^-6^ | -0.06 (-0.19, 0.07) *P* = 0.372 | 0.06 (-0.08, 0.21) *P* = 0.392 | -0.03 (-0.11, 0.06)  *P* = 0.539 |
| No access to a garden  (2% /18% / 8%) | 0.47 (0.09, 0.85) *P* = 0.016 | 0.16 (0.07, 0.24) *P* = 0.0002 | 0.24 (0.12, 0.37)  *P* = 0.0001 | -0.07 (-0.35, 0.21) *P* = 0.62 | 0.05 (-0.04, 0.14) *P* = 0.235 | 0.16 (0.04, 0.28)  *P* = 0.007 |
| Health care worker  (11% / 12% / NA) | 0.01 (-0.09, 0.10) *P* = 0.901 | -0.02 (-0.12, 0.08) *P* = 0.683 | Not assessed | -0.03 (-0.13, 0.07) *P* = 0.597 | 0.02 (-0.08, 0.13) *P* = 0.652 | Not assessed |
| Key worker  (32% / 39% / 22%) | 0.04 (-0.03, 0.11) *P* = 0.214 | -0.09 (-0.15, -0.02) *P* = 0.008 | -0.05 (-0.13, 0.02)  *P* = 0.178 | 0.03 (-0.04, 0.10) *P* = 0.441 | 0.02 (-0.05, 0.09) *P* = 0.631 | 0.04 (-0.03, 0.12)  *P* = 0.266 |
| **Psychiatric or mental health factors** |  |  |  |  |  |  |
| Probable MDD  (8% / 14% / 14%) | 0.38 (0.22, 0.54) *P* = 2.29 x 10^-6^ | 0.31 (0.20, 0.42) *P* = 3.18 x 10^-8^ | 0.39 (0.29, 0.49)  *P* = 1.84 x 10^-13^ | 0.26 (0.11, 0.40) *P* = 0.0005 | 0.49 (0.39, 0.62) *P* = 1.33 x 10^-16^ | 0.27 (0.17, 0.38)  *P* = 7.03 x 10^-7^ |
| Probable GAD  (7% / 13% / Na) | 0.26 (0.11, 0.42) *P* = 0.001 | 0.14 (0.03, 0.25) *P* = 0.010 | Not assessed | 0.25 (0.09, 0.40) *P* = 0.002 | 0.50 (0.39, 0.62) *P* = 2.72 x 10^-17^ | Not assessed |
| Psychosis like experiences  (Na / 15% / scale) | Not assessed | 0.17 (0.07, 0.27) *P* = 0.001 | 0.15 (0.11, 0.19)  *P* = 3.72 x 10^-14^ | Not assessed | 0.25 (0.15, 0.36) *P* = 4.74 x 10^-6^ | 0.12 (0.08, 0.16)  *P* = 9.59 x 10^-9^ |
| Disordered eating  (3% / 9% / Na) | 0.09 (-0.14, 0.32) *P* = 0.689 | 0.21 (0.09, 0.34) *P* = 0.0005 | Not assessed | 0.08 (-0.16, 0.32) *P* = 0.510 | 0.26 (0.12, 0.40) *P* = 0.0002 | Not assessed |
| OCD traits  (scale variable) | Not assessed | 0.05 (0.01, 0.09) *P* = 0.027 | Not assessed | Not assessed | 0.15 (0.11, 0.19) *P* = 8.28 x 10^-13^ | Not assessed |
| Autistic traits  (Na / 7% / Na) | Not assessed | 0.19 (0.05, 0.34) *P* = 0.008 | Not assessed | Not assessed | 0.35 (0.20, 0.51) *P* = 5.18 x 10^-6^ | Not assessed |
| Personality disorder traits  (11% /11% / Na) | 0.32 (0.19, 0.45) *P* = 1.67 x 10^-6^ | 0.09 (-0.04, 0.23) *P* = 0.169 | Not assessed | 0.15 (0.02, 0.27) *P* = 0.021 | 0.27 (0.14, 0.40) *P* = 0.00008 | Not assessed |
| History of alcohol misuse  (17% /9% / 16%) | 0.04 (-0.05, 0.13) *P* = 0.367 | 0.13 (0.01, 0.25) *P* = 0.040 | 0.02 (-0.06, 0.10)  *P* = 0.598 | 0.09 (0.00, 0.18) *P* = 0.047 | 0.20 (0.07, 0.33) *P* = 0.003 | -0.02 (-0.10, 0.06)  *P* = 0.569 |
| Current smokers (tobacco)  (29%/ 12% /10%) | 0.18 (0.10, 0.25) *P* = 7.58 x 10^-6^ | 0.02 (-0.09, 0.13) *P* = 0.690 | 0.30 (0.19, 0.41)  *P* = 1.23 x 10^-7^ | 0.12 (0.05, 0.20) *P* = 0.001 | 0.10 (-0.01, 0.21) *P* = 0.085 | 0.18 (0.08, 0.29)  *P* = 0.001 |
| Negative cognitive styles  (scale variable) | 0.21 (0.16, 0.25) *P* = 1.07 x 10^-18^ | 0.09 (0.05, 0.13) *P* = 0.00004 | 0.22 (0.19, 0.26)  *P* = 2.97 x 10^-32^ | 0.16 (0.12, 0.20) *P* = 3.07 x 10^-14^ | 0.07 (0.02, 0.12) *P* = 0.003 | 0.19 (0.15, 0.22)  *P* = 5.39 x 10^-21^ |
| Difficulties accessing mental health info  (Na / 23% / Na) | Not assessed | 0.12 (0.03, 0.20) *P* = 0.009 | Not assessed | Not assessed | 0.28 (0.19, 0.36) *P* = 1.93 x 10^-9^ | Not assessed |
| Higher neuroticism  (scale variable) | Not assessed | 0.04 (0.01, 0.09) *P* = 0.015 | 0.22 (0.19, 0.26)  *P* = 3.00 x 10^-42^ | Not assessed | 0.11 (0.07, 0.15) *P* = 7.33 x 10^-7^ | 0.21 (0.18, 0.25)  *P* = 1.97 x 10^-31^ |
| Self-harm history  (Na / 24% / 2%) | Not assessed | 0.15 (0.06, 0.23) *P* = 0.001 | 0.55 (0.22, 0.88)  *P* = 0.001 | Not assessed | 0.19 (0.09, 0.28) *P* = 0.0002 | 0.58 (0.28, 0.88)  *P* = 1.97 x 10^-8^ |
| Depression PRS***  (scale variable) | 0.09 (0.05, 0.13) *P* = 0.00002  (n=1906) | 0.03 (-0.02, 0.07) *P* = 0.224  (n=1592) | 0.05 (0.02, 0.08)  *P* = 0.0002  (n=3849) | 0.09 (0.05, 0.14) *P* = 0.00004  (n=2071) | 0.00 (-0.05, 0.05) *P* = 0.993  (n=1329) | 0.06 (0.03, 0.09)  *P* = 0.00002  (n=3832) |

**Supplement table 8.** Associations between pre-pandemic risk factors and depression and anxiety using the imputed samples. Results are standardised estimates for depression and anxiety, adjusted for prior depression or anxiety, sex, age and when the COVID-19 questionnaire was completed. *Indicates the % of individuals with ‘caseness’ within ALSPAC-parents / ALSPAC-young / GS respectively. **Indicates a continuous scale was used so no caseness is given. ***Indicates this was on complete case analysis only. MDD: major depressive disorder; GAD: generalised anxiety disorder; OCD: obsessive compulsive disorder; PRS: polygenic risk score.

|  | **Depression standardised estimates - *β*_STD_ (95% CIs), *P*** | | | **Anxiety standardised estimates - *β*_STD_ (95% CIs), *P*** | | |
| --- | --- | --- | --- | --- | --- | --- |
|  | **ALSPAC-parents** | **ALSPAC-young** | **Gen Scot** | **ALSPAC-parents** | **ALSPAC-young** | **Gen Scot** |
| **Sociodemographic factors** |  |  |  |  |  |  |
| Sex (female)  (76% / 72% / 64%)* | 0.30 (0.24, 0.37) *P* = 1.46 x 10^-19^ | 0.27 (0.19, 0.36) *P* = 1.62 x 10^-9^ | 0.26 (0.20, 0.31)  *P* = 4.10 x 10^-57^ | 0.41 (0.35, 0.48) *P* = 8.81 x 10^-37^ | 0.43 (0.33, 0.53) *P* = 4.58 x 10^-17^ | 0.18 (0.12, 0.23)  *P* = 8.31 x 10^-44^ |
| Age (older ages)  (scale variable)** | -0.02 (-0.03, -0.01) *P* = 0.00002 | -0.01 (-0.08, 0.07) *P* = 0.858 | -0.02 (-0.02, -0.02) *P* = 9.28 x 10^-57^ | -0.02 (-0.03, -0.02) *P* = 1.84 x 10^-10^ | -0.01 (-0.09, 0.08) *P* = 0.866 | -0.02 (-0.02, -0.01) *P* = 4.07 x 10^-47^ |
| Lower educational background  (11%/ 16% / 13%) | 0.22 (0.09, 0.35) *P* = 0.001 | 0.05 (-0.06, 0.17) *P* = 0.167 | 0.07 (0.03, 0.11) *P* = 0.0002 | 0.16 (0.03, 0.28) *P* = 0.013 | 0.12 (-0.01, 0.26) *P* = 0.070 | 0.05 (0.01, 0.09) *P* = 0.018 |
| Higher income  (scale variable) | -0.04 (-0.05, -0.02) *P* = 3.09 x 10^-7^ | -0.01 (-0.04, 0.02) *P* = 0.492 | -0.10 (-0.13, -0.08)  *P* = 5.33 x 10^-19^ | -0.02 (-0.03, -0.01) *P* = 0.048 | -0.02 (-0.05, 0.02) *P* = 0.366 | -0.06 (-0.08, -0.04)  *P* = 2.40 x 10^-7^ |
| Worse neighbourhood area deprivation  (scale variable) | 0.07 (0.03, 0.10) *P* = 0.00004 | 0.00 (-0.03, 0.03) *P* = 0.992 | 0.07 (0.03, 0.11)  *P* = 4.35 x 10^-9^ | 0.06 (0.03, 0.09) *P* = 0.0002 | 0.04 (0.01, 0.08) *P* = 0.034 | 0.04 (0.02, 0.07)  *P* = 0.0001 |
| Financial problems (binary)  (11% / 10% / 4%) | 0.20 (0.06, 0.34) *P* = 0.005 | 0.20 (0.06, 0.34) *P* = 0.005 | 0.38 (0.23, 0.52)  *P* = 8.81 x 10^-7^ | 0.17 (0.04, 0.30) *P* = 0.011 | 0.20 (0.03, 0.36) *P* = 0.021 | 0.19 (0.04, 0.35)  *P* = 0.014 |
| Partner emotional abuse  (8% / Na / Na) | 0.33 (0.16, 0.51) *P* = 0.00019 | Not assessed | Not assessed | 0.28 (0.10, 0.46) *P* = 0.003 | Not assessed | Not assessed |
| Parent with young children  (Na / 11% / 11%) | Not assessed | 0.03 (-0.09, 0.16) *P* = 0.558 | -0.05 (-0.14, 0.05)  *P* = 0.363 | Not assessed | 0.34 (0.19, 0.48) *P* = 3.61 x 10^-6^ | 0.05 (-0.05, 0.16)  *P* = 0.291 |
| **Physical health factors** |  |  |  |  |  |  |
| Obesity  (18 % / 14% / 20%) | 0.18 (0.07, 0.30) *P* = 0.001 | 0.20 (0.07, 0.32) *P* = 0.003 | 0.15 (0.12, 0.19)  *P* = 1.35 x 10^-17^ | 0.20 (0.05, 0.14) *P* = 0.00005 | 0.11 (-0.03, 0.24)† *P* = 0.121 | 0.05 (0.01, 0.08)  *P* = 0.009 |
| Asthma  (16% / 10% / 10%) | 0.07 (-0.04, 0.19) *P* = 0.211 | 0.09 (-0.05, 0.24) *P* = 0.216 | 0.19 (0.10, 0.28)  *P* = 3.04 x 10^-5^ | 0.17 (0.04, 0.29) *P* = 0.0009 | 0.19 (0.03, 0.26) *P* = 0.018 | 0.13 (0.04, 0.22)  *P* = 0.006 |
| **COVID-19 specific risk factors** |  |  |  |  |  |  |
| COVID-19 infection  (12% /16% / 8%) | 0.17 (0.05, 0.28) *P* = 0.004 | 0.10 (-0.01, 0.20) *P* = 0.079 | 0.19 (0.10, 0.29)  *P* = 0.0001 | 0.20 (0.08, 0.32) *P* = 0.001 | 0.09 (-0.03, 0.21) *P* = 0.145 | 0.12 (0.02, 0.22)†  *P* = 0.021 |
| Self-isolation  (19% /25% / Na) | 0.19 (0.10, 0.29) *P* = 0.00009 | 0.12 (0.03, 0.21) *P* = 0.011 | Not assessed | 0.16 (0.07, 0.26) *P* = 0.001 | 0.17 (0.06, 0.27) *P* = 0.002 | Not assessed |
| Living alone  (8% / 6% / 16%) | 0.46 (0.30, 0.61) *P* = 1.89 x 10^-8^ | 0.21 (0.04, 0.37) *P* = 0.015 | 0.21 (0.13, 0.28)  *P* = 2.76 x 10^-8^ | -0.06 (-0.19, 0.07) *P* = 0.382 | -0.04 (-0.23, 0.14) *P* = 0.636 | -0.03 (-0.10, 0.05)  *P* = 0.511 |
| No access to a garden  (2% /18% / 8%) | 0.29 (-0.10, 0.68)† *P* = 0.150 | 0.16 (0.06, 0.26) *P* = 0.002 | 0.28 (0.18, 0.38)  *P* = 3.37 x 10^-08^ | 0.00 (-0.33, 0.33) *P* = 0.987 | 0.03 (-0.09, 0.14) *P* = 0.642 | 0.18 (0.08, 0.29)  *P* = 0.0004 |
| Health care worker  (11% / 12% / NA) | -0.04 (-0.14, 0.06) *P* = 0.428 | -0.01 (-0.14, 0.11) *P* = 0.827 | Not assessed | -0.01 (-0.12, 0.09) *P* = 0.832 | 0.03 (-0.11, 0.16) *P* = 0.722 | Not assessed |
| Key worker  (32% / 39% / 22%) | 0.05 (-0.03, 0.12) *P* = 0.244 | -0.09 (-0.17, -0.01) *P* = 0.025 | -0.06 (-0.13, 0.01)  *P* = 0.118 | 0.03 (-0.05, 0.11) *P* = 0.482 | -0.01 (-0.10, 0.09) *P* = 0.903 | 0.04 (-0.03, 0.11)  *P* = 0.311 |
| **Psychiatric or mental health risk factors** |  |  |  |  |  |  |
| Probable MDD  (8% / 14% / 14%) | 0.37 (0.20, 0.54) *P* = 0.00001 | 0.36 (0.23, 0.48) *P* = 4.94 x 10^-8^ | 0.37 (0.29, 0.46)  *P* = 1.67 x 10^-19^ | 0.25 (0.09, 0.41) *P* = 0.002 | 0.53 (0.19, 0.66) *P* = 9.83 x 10^-14^ | 0.29 (0.21, 0.37)  *P* = 9.37 x 10^-12^ |
| Probable GAD  (7% / 13% / Na) | 0.21 (0.02, 0.40) *P* = 0.031 | 0.20 (0.07, 0.33) *P* = 0.003 | Not assessed | 0.25 (0.08, 0.35) *P* = 0.004 | 0.51 (0.37, 0.65) *P* = 6.03 x 10^-12^ | Not assessed |
| Psychosis like experiences  (Na / 15% / scale) | Not assessed | 0.20 (0.08, 0.32) *P* = 0.001 | 0.17 (0.13, 0.21)  *P* = 3.29 x 10^-18^ | Not assessed | 0.27 (0.14, 0.41) *P* = 0.00006 | 0.14 (0.10, 0.18)  *P* = 1.13 x 10^-12^ |
| Disordered eating  (3% / 9% / Na) | 0.05 (-0.19, 0.29) *P* = 0.689 | 0.22 (0.07, 0.37) *P* = 0.003 | Not assessed | 0.08 (-0.18, 0.35) *P* = 0.544 | 0.20 (0.04, 0.37) *P* = 0.014 | Not assessed |
| OCD traits  (scale variable) | Not assessed | 0.05 (0.01, 0.09) *P* = 0.049 | Not assessed | Not assessed | 0.17 (0.12, 0.23) *P* = 6.02 x 10^-10^ | Not assessed |
| Autistic traits  (Na / 7% / Na) | Not assessed | 0.21 (0.04, 0.37) *P* = 0.014 | Not assessed | Not assessed | 0.33 (0.14, 0.52) *P* = 0.001 | Not assessed |
| Personality disorder traits  (11% /11% / Na) | 0.32 (0.18, 0.46) *P* = 7.87 x 10^-6^ | 0.15 (0.01, 0.29)† *P* = 0.044 | Not assessed | 0.15 (0.02, 0.28) *P* = 0.02 | 0.32 (0.17, 0.48) *P* = 0.00006 | Not assessed |
| History of alcohol misuse  (17% /9% / 16%) | 0.03 (-0.06, 0.12) *P* = 0.521 | 0.16 (0.02, 0.30) *P* = 0.029 | 0.01 (-0.07, 0.09)  *P* = 0.748 | 0.09 (-0.01, 0.18) *P* = 0.090 | 0.23 (0.07, 0.39) *P* = 0.004 | -0.02 (-0.10, 0.06)  *P* = 0.667 |
| Current smokers (tobacco)  (29%/ 12% /10%) | 0.16 (0.09, 0.24) *P* = 0.00004 | 0.06 (-0.07, 0.19) *P* = 0.357 | 0.30 (0.22, 0.39)  *P* = 1.74 x 10^-11^ | 0.14 (0.05, 0.22) *P* = 0.001 | 0.18 (0.03, 0.33)† *P* = 0.019 | 0.19 (0.10, 0.28)  *P* = 0.00003 |
| Negative cognitive styles  (scale variable) | 0.19 (0.24, 0.37) *P* = 5.21 x 10^-17^ | 0.10 (0.06, 0.15) *P* = 0.00002 | 0.23 (0.19, 0.26)  *P* = 3.23 x 10^-42^ | 0.17 (0.13, 0.22) *P* = 1.24 x 10^-12^ | 0.09 (0.03, 0.14) *P* = 0.001 | 0.19 (0.16, 0.2)  *P* = 1.42 x 10^-28^ |
| Difficulties accessing mental health info  (Na / 23% / Na) | Not assessed | 0.14 (0.04, 0.23) *P* = 0.006 | Not assessed | Not assessed | 0.32 (0.21, 0.43) *P* = 1.03 x 10^-8^ | Not assessed |
| Higher neuroticism  (scale variable) | Not assessed | 0.04 (0.01, 0.08) *P* = 0.034 | 0.24 (0.21, 0.27)  *P* = 3.30 x 10^-56^ | Not assessed | 0.10 (0.05, 0.14) *P* = 0.00009 | 0.24 (0.21, 0.27)  *P* = 1.12 x 10^-48^ |
| History of self-harm  (Na / 24% / 2%) | Not assessed | 0.16 (0.07, 0.35) *P* = 0.0004 | 0.64 (0.42, 0.86)  *P* = 1.46 x 10^-8^ | Not assessed | 0.17 (0.07, 0.28) *P* = 0.001 | 0.65 (0.42, 0.88)  *P* = 1.97 x 10^-8^ |
| Depression PRS  (scale variable) | 0.09 (0.05, 0.13) *P* = 0.00002 | 0.03 (-0.02, 0.07) *P* = 0.224 | 0.05 (0.02, 0.08)  *P* = 0.0002 | 0.09 (0.05, 0.14) *P* = 0.00004 | 0.00 (-0.05, 0.05) *P* = 0.993 | 0.06 (0.03, 0.09)  *P* = 0.00002 |

**Supplement table 9.** Associations between pre-pandemic risk factors and depression and anxiety during COVID-19 using the complete case samples in ALSPAC-parents, ALSPAC-young and Generation Scotland. Results show standardised estimates, adjusted for prior depression or anxiety (most recent pre-pandemic assessment), sex, age and when the COVID-19 questionnaire was completed. *Indicates the % of individuals with caseness for ALSPAC-parents / ALSPAC-young / GS respectively. **Indicates a continuous scale was used so no proportions are given. †Indicates a substantive difference from the imputed results. MDD: major depressive disorder; GAD: generalised anxiety disorder; OCD: obsessive compulsive disorder; PRS: polygenic risk score.

|  | **Depression standardised estimates - *β*_STD_ (95% CIs), *P*** | | | **Anxiety standardised estimates - *β*_STD_ (95% CIs), *P*** | | |
| --- | --- | --- | --- | --- | --- | --- |
|  | **ALSPAC-parents**  **(n=3579)** | **ALSPAC-young**  **(n=2872)** | **Gen Scot**  **(n=4208)** | **ALSPAC-parents**  **(n=3579)** | **ALSPAC-young**  **(n=2872)** | **Gen Scot**  **(n=4208)** |
| **Sociodemographic factors** |  |  |  |  |  |  |
| Sex (female)  (76% / 72% / 64%)* | 0.27 (0.21, 0.34) *P* = 3.67 x 10^-16^ | 0.22 (0.15, 0.29) *P* = 1.97 x 10^-9^ | 0.27 (0.21, 0.32)  *P* = 1.01 x 10^-21^ | 0.25 (0.20, 0.32) *P* = 4.44 x 10^-16^ | 0.40 (0.32, 0.47) *P* = 2.70 x 10^-22^ | 0.17 (0.12, 0.23)  *P* = 8.11 x 10^-10^ |
| Age (older ages)  (scale variable)** | -0.02 (-0.02, -0.01) *P* = 2.22 x 10^-6^ | -0.01 (-0.07, 0.04) *P* = 0.646 | -0.02 (-0.02, -0.02) *P* = 1.53 x 10^-44^ | -0.02 (-0.03, -0.01) *P* = 1.40 x 10^-9^ | 0.00 (-0.06, 0.07) *P* = 0.936 | -0.01 (-0.02, -0.01) *P* = 1.56 x 10^-29^ |
| Lower educational background  (11%/ 16% / 13%) | 0.23 (0.12, 0.35) *P* = 0.00008 | 0.05 (-0.04, 0.15) *P* = 0.299 | 0.15 (0.06, 0.24) *P* = 0.001 | 0.14 (0.03, 0.25) *P* = 0.016 | 0.16 (0.06, 0.27) *P* = 0.002 | 0.09 (0.00, 0.18) *P* = 0.061 |
| Higher income  (scale variable) | -0.04 (-0.05, -0.02) *P* = 1.52 x 10^-6^ | -0.01 (-0.04, 0.01) *P* = 0.365 | -0.10 (-0.12, -0.08)  *P* = 2.02 x 10^-13^ | -0.01 (-0.02, 0.00)† *P* = 0.151 | -0.04 (-0.06, -0.01) *P* = 0.017 | -0.06 (-0.09, -0.03)  *P* = 5.73 x 10^-6^ |
| Worse deprivation status (scale variable) | 0.06 (0.03, 0.09) *P* = 0.00009 | 0.01 (-0.02, 0.04) *P* = 0.404 | 0.06 (0.03, 0.08)  *P* = 8.38 x 10^-6^ | 0.05 (0.02, 0.08) *P* = 0.002 | 0.04 (0.01, 0.07) *P* = 0.025 | 0.04 (0.01, 0.06)  *P* = 0.004 |
| Financial problems  (11% / 10% / 4%) | 0.28 (0.14, 0.42) *P* = 0.00009 | 0.14 (0.02, 0.25) *P* = 0.018 | 0.38 (0.18, 0.58)  *P* = 0.0002 | 0.19 (0.07, 0.32) *P* = 0.003 | 0.22 (0.10, 0.35) *P* = 0.0004 | 0.20 (0.00, 0.39)  *P* = 0.049 |
| Partner emotional abuse  (8% / Na / Na) | 0.36 (0.18, 0.53) *P* = 0.00006 | Not assessed | Not assessed | 0.29 (0.13, 0.46) *P* = 0.001 | Not assessed | Not assessed |
| Parent with young children  (Na / 11% / 11%) | Not assessed | 0.02 (-0.09, 0.12) *P* = 0.733 | -0.01 (-0.12, 0.10)  *P* = 0.811 | Not assessed | 0.16 (0.05, 0.27) *P* = 0.004 | 0.06 (-0.05, 0.17)  *P* = 0.258 |
| **Physical health factors** |  |  |  |  |  |  |
| Obesity  (18 % / 14% / 20%) | 0.22 (0.12, 0.32) *P* = 0.00003 | 0.18 (0.05, 0.30) *P* = 0.005 | 0.31 (0.28, 0.65)  *P* = 1.07 x 10^-13^ | 0.14 (0.04, 0.24) *P* = 0.008 | 0.13 (0.01, 0.25) *P* = 0.039 | 0.09 (0.02, 0.17)  *P* = 0.018 |
| Asthma  (16% / 10% / 10%) | 0.08 (-0.01, 0.17) *P* = 0.092 | 0.08 (-0.04, 0.20) *P* = 0.203 | 0.17 (0.07, 0.28)  *P* = 0.001 | 0.07 (-0.02, 0.16) *P* = 0.117 | 0.20 (0.07, 0.34) *P* = 0.004 | 0.12 (0.02, 0.22)  *P* = 0.018 |
| **COVID-19 specific factors** |  |  |  |  |  |  |
| COVID-19 infection  (12% /16% / 8%) | 0.18 (0.07, 0.28) *P* = 0.001 | 0.09 (0.00, 0.17) *P* = 0.045 | 0.18 (0.06, 0.30)  *P* = 0.003 | 0.16 (0.06, 0.27) *P* = 0.002 | 0.08 (-0.02, 0.17) *P* = 0.106 | 0.10 (-0.01, 0.22)  *P* = 0.079 |
| Self-isolation  (19% /25% / Na) | 0.20 (0.11, 0.28) *P* = 0.00001 | 0.15 (0.08, 0.22) *P* = 0.00004 | Not assessed | 0.13 (0.04, 0.21) *P* = 0.004 | 0.17 (0.09, 0.25) *P* = 0.00003 | Not assessed |
| Living alone  (8% / 6% / 16%) | 0.45 (0.30, 0.60) *P* = 7.07 x 10^-9^ | 0.20 (0.06, 0.34) *P* = 0.005 | 0.19 (0.11, 0.27)  *P* = 6.36 x 10^-6^ | -0.06 (-0.18, 0.07) *P* = 0.353 | 0.06 (-0.09, 0.21) *P* = 0.437 | -0.03 (-0.11, 0.05)  *P* = 0.501 |
| No access to a garden  (2% /18% / 8%) | 0.48 (0.10, 0.86) *P* = 0.014 | 0.16 (0.08, 0.24) *P* = 0.0002 | 0.23 (0.11, 0.35)  *P* = 0.0003 | -0.06 (-0.34, 0.21) *P* = 0.658 | 0.06 (-0.03, 0.15) *P* = 0.168 | 0.15 (0.04, 0.27)  *P* = 0.012 |
| Health care worker  (11% / 12% / NA) | 0.02 (-0.08, 0.11) *P* = 0.727 | -0.02 (-0.12, 0.08) *P* = 0.693 | Not assessed | -0.02 (-0.12, 0.08) *P* = 0.746 | 0.03 (-0.08, 0.13) *P* = 0.613 | Not assessed |
| Key worker  (32% / 39% / 22%) | 0.03 (-0.04, 0.10) *P* = 0.334 | -0.09 (-0.16, -0.03) *P* = 0.005 | -0.05 (-0.12, 0.03)  *P* = 0.192 | 0.02 (-0.05, 0.09) *P* = 0.549 | 0.01 (-0.07, 0.08) *P* = 0.894 | 0.05 (-0.03, 0.12)  *P* = 0.245 |
| **Psychiatric or mental health risk factors** |  |  |  |  |  |  |
| Probable MDD  (8% / 14% / 14%) | 0.37 (0.22, 0.53) *P* = 3.68 x 10^-6^ | 0.31 (0.20, 0.42) *P* = 3.71 x 10^-8^ | 0.39 (0.29, 0.49)  *P* = 1.74 x 10^-13^ | 0.26 (0.11, 0.40) *P* = 0.0005 | 0.49 (0.37, 0.60) *P* = 4.06 x 10^-16^ | 0.28 (0.17, 0.39)  *P* = 5.07 x 10^-7^ |
| Probable GAD  (7% / 13% / Na) | 0.26 (0.10, 0.42) *P* = 0.001 | 0.14 (0.04, 0.25) *P* = 0.010 | Not assessed | 0.25 (0.09, 0.40) *P* = 0.002 | 0.50 (0.39, 0.62) *P* = 3.13 x 10^-16^ | Not assessed |
| Psychosis like experiences  (Na / 15% / scale) | Not assessed | 0.17 (0.06, 0.27) *P* = 0.001 | 0.15 (0.11, 0.18)  *P* = 3.72 x 10^-14^ | Not assessed | 0.24 (0.14, 0.35) *P* = 0.00001 | 0.12 (0.08, 0.16)  *P* = 1.85 x 10^-8^ |
| Disordered eating  (3% / 9% / Na) | 0.10 (-0.13, 0.33) *P* = 0.395 | 0.21 (0.09, 0.33) *P* = 0.001 | Not assessed | 0.09 (-0.15, 0.33) *P* = 0.454 | 0.25 (0.11, 0.38) *P* = 0.0003 | Not assessed |
| OCD traits  (scale variable) | Not assessed | 0.05 (0.01, 0.09) *P* = 0.031 | Not assessed | Not assessed | 0.15 (0.11, 0.19) *P* = 6.03 x 10^-12^ | Not assessed |
| Autistic traits  (Na / 7% / Na) | Not assessed | 0.19 (0.05, 0.34) *P* = 0.009 | Not assessed | Not assessed | 0.34 (0.19, 0.49) *P* = 0.00001 | Not assessed |
| Personality disorder traits  (11% /11% / Na) | 0.30 (0.17, 0.43) *P* = 5.20 x 10^-6^ | 0.09 (-0.04, 0.23) *P* = 0.177 | Not assessed | 0.14 (0.01, 0.26) *P* = 0.032 | 0.26 (0.12, 0.39) *P* = 0.0002 | Not assessed |
| History of alcohol misuse  (17% /9% / 16%) | 0.04 (-0.05, 0.13) *P* = 0.353 | 0.13 (0.01, 0.25) *P* = 0.036 | 0.01 (-0.06, 0.09)  *P* = 0.716 | 0.09 (0.00, 0.18) *P* = 0.046 | 0.20 (0.08, 0.33) *P* = 0.002 | -0.03 (-0.11, 0.05)  *P* = 0.494 |
| Current smokers (tobacco)  (29%/ 12% /10%) | 0.16 (0.08, 0.24) *P* = 0.00006 | 0.02 (-0.09, 0.13) *P* = 0.747 | 0.28 (0.17, 0.39)  *P* = 8.66 x 10^-7^ | 0.11 (0.04, 0.19) *P* = 0.004 | 0.08 (-0.03, 0.20) *P* = 0.149 | 0.17 (0.07, 0.28)  *P* = 0.002 |
| Negative cognitive styles  (scale variable) | 0.21 (0.17, 0.26) *P* = 9.94 x 10^-20^ | 0.09 (0.05, 0.13) *P* = 0.00004 | 0.22 (0.19, 0.26)  *P* = 1.31 x 10^-31^ | 0.16 (0.12, 0.20) *P* = 5.27 x 10^-15^ | 0.07 (0.03, 0.12) *P* = 0.003 | 0.19 (0.15, 0.22)  *P* = 7.13 x 10^-21^ |
| Difficulties accessing mental health info  (Na / 23% / Na) | Not assessed | 0.12 (0.03, 0.20) *P* = 0.008 | Not assessed | Not assessed | 0.28 (0.19, 0.37) *P* = 9.72 x 10^-10^ | Not assessed |
| Higher neuroticism  (scale variable) | Not assessed | 0.05 (0.01, 0.09) *P* = 0.015 | 0.22 (0.19, 0.25)  *P* = 3.00 x 10^-40^ | Not assessed | 0.11 (0.07, 0.15) *P* = 1.35 x 10^-6^ | 0.21 (0.18, 0.25)  *P* = 1.10 x 10^-30^ |
| History of self-harm  (Na / 24% / 2%) | Not assessed | 0.15 (0.06, 0.24) *P* = 0.001 | 0.53 (0.20, 0.86)  *P* = 0.002 | Not assessed | 0.19 (0.09, 0.29) *P* = 0.0002 | 0.57 (0.27, 0.87)  *P* = 0.0002 |
| Depression PRS  (scale variable) | 0.09 (0.05, 0.13) *P* = 0.00002  (n=1906) | 0.03 (-0.02, 0.07) *P* = 0.224  (n=1592) | 0.05 (0.02, 0.08)  *P* = 0.0002  (n=3849) | 0.09 (0.05, 0.14) *P* = 0.00004  (n=2071) | 0.00 (-0.05, 0.05) *P* = 0.993  (n=1329) | 0.06 (0.03, 0.09)  *P* = 0.00002  (n=3832) |

**Supplement table 10.** Associations between pre-pandemic risk factors and depression and anxiety, adjusting for educational background using the imputed samples in ALSPAC-parents, ALSPAC-young and Generation Scotland. Results are standardised estimates adjusted for depression or anxiety (most recent pre-pandemic assessment), sex, age and when the COVID-19 questionnaire was completed. *Indicates the % of individuals with caseness for ALSPAC-parents / ALSPAC-young / GS respectively. ** Indicates a continuous scale was used so no proportions are given. †Indicates a substantive difference from the imputed results. MDD: major depressive disorder; GAD: generalised anxiety disorder; OCD: obsessive compulsive disorder; PRS: polygenic risk score.

|  | **Depression standardised estimates - *β*_STD_ (95% CIs), *P*** | | | | |
| --- | --- | --- | --- | --- | --- |
|  | **Imputed SMFQ pre-pandemic assessment: Age 26** | **Complete case SMFQ**  **pre-pandemic assessment: Age 26** | **Imputed SMFQ education adjusted**  **pre-pandemic assessment: Age 26** | **Complete case SMFQ pre-pandemic assessment: Age 24** | **Complete case SMFQ**  **pre-pandemic assessment: Age 22** |
| **Sociodemographic factors** |  |  |  |  |  |
| Sex (female)  (72%)* | 0.22 (0.15, 0.29) *P* = 1.20 x 10^-9^ | 0.27 (0.19, 0.36) *P* = 1.62 x 10^-9^ | 0.22 (0.15, 0.29) *P* = 1.97 x 10^-9^ | 0.28 (0.19, 0.36) *P* = 1.07 x 10^-10^ | 0.32 (0.23, 0.41) *P* = 2.54 x 10^-11^ |
| Age (older ages)  (scale variable)** | -0.01 (-0.07, 0.04) *P* = 0.643 | -0.01 (-0.08, 0.07) *P* = 0.858 | -0.01 (-0.07, 0.04) *P* = 0.646 | -0.02 (-0.09, 0.05) *P* = 0.579 | -0.01 (-0.09, 0.06) *P* = 0.730 |
| Lower educational background  (16%) | 0.05 (-0.04, 0.15) *P* = 0.276 | 0.05 (-0.06, 0.17) *P* = 0.167 | 0.05 (-0.04, 0.15) *P* = 0.299 | 0.10 (-0.01, 0.21) *P* = 0.072 | 0.14 (0.02, 0.27)† *P* = 0.026 |
| Higher income  (scale variable) | -0.01 (-0.04, 0.01) *P* = 0.328 | -0.01 (-0.04, 0.02) *P* = 0.492 | -0.01 (-0.04, 0.01) *P* = 0.365 | -0.02 (-0.05, 0.01) *P* = 0.148 | -0.02 (-0.05, 0.02) *P* = 0.293 |
| Worse deprivation status (scale variable) | 0.01 (-0.01, 0.04) *P* = 0.319 | 0.00 (-0.03, 0.03) *P* = 0.992 | 0.01 (-0.02, 0.04) *P* = 0.404 | 0.03 (0.00, 0.07)† *P* = 0.027 | 0.04 (0.00, 0.08)† *P* = 0.027 |
| Financial problems  (10%) | 0.14 (0.03, 0.26) *P* = 0.015 | 0.20 (0.06, 0.34) *P* = 0.005 | 0.14 (0.02, 0.25) *P* = 0.018 | 0.36 (0.22, 0.50) *P* = 2.67 x 10^-7^ | 0.26 (0.11, 0.42) *P* = 0.001 |
| Partner emotional abuse  (Na) | Not assessed | Not assessed | Not assessed | Not assessed | Not assessed |
| Parent with young children  (11%) | 0.03 (-0.07, 0.13) *P* = 0.570 | 0.03 (-0.09, 0.16) *P* = 0.558 | 0.02 (-0.09, 0.12) *P* = 0.733 | 0.14 (0.02, 0.25)† *P* = 0.020 | 0.16 (0.03, 0.30)† *P* = 0.019 |
| **Physical health factors** |  |  |  |  |  |
| Obesity  (14%) | 0.18 (0.06, 0.31) *P* = 0.004 | 0.20 (0.07, 0.32) *P* = 0.003 | 0.18 (0.05, 0.30) *P* = 0.005 | 0.20 (0.08, 0.32) *P* = 0.001 | 0.25 (0.11, 0.38) *P* = 0.0003 |
| Asthma  (10%) | 0.08 (-0.04, 0.20) *P* = 0.196 | 0.09 (-0.05, 0.24) *P* = 0.216 | 0.08 (-0.04, 0.20) *P* = 0.203 | 0.05 (-0.08, 0.18) *P* = 0.490 | 0.10 (-0.05, 0.24) *P* = 0.191 |
| **COVID-19 specific factors** |  |  |  |  |  |
| COVID-19 infection  (16%) | 0.09 (0.00, 0.17) *P* = 0.045 | 0.10 (-0.01, 0.20) *P* = 0.079 | 0.09 (0.00, 0.17) *P* = 0.045 | 0.06 (-0.04, 0.16)† *P* = 0.208 | 0.03 (-0.09, 0.14)† *P* = 0.645 |
| Self-isolation  (25%) | 0.15 (0.08, 0.22) *P* = 0.00004 | 0.12 (0.03, 0.21) *P* = 0.011 | 0.15 (0.08, 0.22) *P* = 0.00004 | 0.14 (0.05, 0.22) *P* = 0.001 | 0.11 (0.01, 0.21) *P* = 0.001 |
| Living alone  (6%) | 0.20 (0.06, 0.34) *P* = 0.005 | 0.21 (0.04, 0.37) *P* = 0.015 | 0.20 (0.06, 0.34) *P* = 0.005 | 0.22 (0.06, 0.38) *P* = 0.006 | 0.23 (0.06, 0.41) *P* = 0.033 |
| No access to a garden  (18%) | 0.16 (0.07, 0.24) *P* = 0.0002 | 0.16 (0.06, 0.26) *P* = 0.002 | 0.16 (0.08, 0.24) *P* = 0.0002 | 0.12 (0.02, 0.21) *P* = 0.018 | 0.15 (0.05, 0.26) *P* = 0.005 |
| Health care worker  (12%) | -0.02 (-0.12, 0.08) *P* = 0.683 | -0.01 (-0.14, 0.11) *P* = 0.827 | -0.02 (-0.12, 0.08) *P* = 0.693 | 0.02 (-0.10, 0.13) *P* = 0.944 | 0.02 (-0.11, 0.14) *P* = 0.801 |
| Key worker  (39%) | -0.09 (-0.15, -0.02) *P* = 0.008 | -0.09 (-0.17, 0.01) *P* = 0.025 | -0.09 (-0.16, -0.03) *P* = 0.005 | -0.08 (-0.16, 0.00) *P* = 0.040 | -0.08 (-0.17, 0.01) *P* = 0.068 |
| **Psychiatric or mental health factors** |  |  |  |  |  |
| Probable MDD  (14%) | 0.31 (0.20, 0.42) *P* = 3.18 x 10^-8^ | 0.36 (0.23, 0.48) *P* = 4.94 x 10^-8^ | 0.31 (0.20, 0.42) *P* = 3.71 x 10^-8^ | 0.43 (0.31, 0.54) *P* = 2.27 x 10^-13^ | 0.56 (0.43, 0.68) *P* = 3.87 x 10^-17^ |
| Probable GAD  (13%) | 0.14 (0.03, 0.25) *P* = 0.010 | 0.20 (0.07, 0.33) *P* = 0.003 | 0.14 (0.04, 0.25) *P* = 0.010 | 0.23 (0.12, 0.35) *P* = 0.0001 | 0.29 (0.16, 0.42) *P* = 0.00002 |
| Psychosis like experiences  (15%) | 0.17 (0.07, 0.27) *P* = 0.001 | 0.20 (0.08, 0.32) *P* = 0.001 | 0.17 (0.06, 0.27) *P* = 0.001 | 0.28 (0.17, 0.39) *P* = 4.17 x 10^-7^ | 0.28 (0.16, 0.40) *P* = 0.00001 |
| Disordered eating  (9%) | 0.21 (0.09, 0.34) *P* = 0.0005 | 0.22 (0.07, 0.37) *P* = 0.003 | 0.21 (0.09, 0.33) *P* = 0.001 | 0.24 (0.10, 0.38) *P* = 0.001 | 0.28 (0.12, 0.43) *P* = 0.001 |
| OCD traits  (scale variable) | 0.05 (0.01, 0.09) *P* = 0.027 | 0.05 (0.01, 0.09) *P* = 0.049 | 0.05 (0.01, 0.09) *P* = 0.031 | 0.05 (0.01, 0.10) *P* = 0.014 | 0.12 (0.07, 0.17) *P* = 5.37 x 10^-6^ |
| Autistic traits  (7%) | 0.19 (0.05, 0.34) *P* = 0.008 | 0.21 (0.04, 0.37) *P* = 0.014 | 0.19 (0.05, 0.34) *P* = 0.009 | 0.35 (0.19, 0.50) *P* = 0.00001 | 0.35 (0.16, 0.53) *P* = 0.0002 |
| Personality disorder traits  (11%) | 0.09 (0.04, 0.23) *P* = 0.169 | 0.15 (0.01, 0.29)† *P* = 0.044 | 0.09 (-0.04, 0.23) *P* = 0.177 | 0.19 (0.05, 0.32)† *P* = 0.006 | 0.35 (0.20, 0.50)† *P* = 3.92 x 10^-6^ |
| History of alcohol misuse  (9%) | 0.13 (0.01, 0.25) *P* = 0.040 | 0.16 (0.02, 0.30) *P* = 0.029 | 0.13 (0.01, 0.25) *P* = 0.036 | 0.15 (0.01, 0.28) *P* = 0.030 | 0.20 (0.05, 0.35) *P* = 0.009 |
| Current smokers (tobacco)  (12%) | 0.02 (-0.09, 0.13) *P* = 0.690 | 0.06 (-0.07, 0.19) *P* = 0.357 | 0.02 (-0.09, 0.13) *P* = 0.747 | 0.03 (-0.09, 0.15) *P* = 0.639 | 0.17 (0.02, 0.31)† *P* = 0.022 |
| Negative cognitive styles  (scale variable) | 0.09 (0.05, 0.13) *P* = 0.00004 | 0.10 (0.06, 0.15) *P* = 0.00002 | 0.09 (0.05, 0.13) *P* = 0.00004 | 0.09 (0.04, 0.13) *P* = 0.00007 | 0.11 (0.06, 0.16) *P* = 3.57 x 10^-6^ |
| Difficulties accessing mental health info  (23%) | 0.12 (0.03, 0.20) *P* = 0.009 | 0.14 (0.04, 0.23) *P* = 0.006 | 0.12 (0.03, 0.20) *P* = 0.008 | 0.21 (0.12, 0.31) *P* = 0.00001 | 0.30 (0.19, 0.40) *P* = 2.96 x 10^-8^ |
| Higher neuroticism  (scale variable) | 0.04 (0.01, 0.09) *P* = 0.015 | 0.04 (0.01, 0.08) *P* = 0.034 | 0.05 (0.01, 0.09) *P* = 0.015 | 0.07 (0.02, 0.11) *P* = 0.003 | 0.07 (0.03, 0.12) *P* = 0.002 |
| History of self-harm  (24%) | 0.15 (0.06, 0.23) *P* = 0.001 | 0.16 (0.07, 0.25) *P* = 0.0004 | 0.15 (0.06, 0.24) *P* = 0.001 | 0.17 (0.07, 0.26) *P* = 0.001 | 0.19 (0.09, 0.29) *P* = 0.0004 |
| Depression PRS  (scale variable) | 0.03 (-0.02, 0.07) *P* = 0.224 | 0.03 (-0.02, 0.07) *P* = 0.224 | 0.03 (-0.02, 0.07) *P* = 0.224 | 0.01 (-0.03, 0.05) *P* = 0.649 | 0.05 (0.00, 0.09) *P* = 0.061 |

**Supplement table 11.** Association between pre-pandemic risk factors and depression during COVID-19 with imputation and complete case data at different pre-pandemic timings in ALSPAC-young. Results are standardised estimates adjusted for prior depression (at the stated pre-pandemic assessment), sex, age and when the COVID-19 questionnaire was completed. *Indicates the % of individuals with caseness for ALSPAC-parents / ALSPAC-young / GS respectively. **Indicates a continuous scale was used so no proportions are given. †Indicates a substantive difference from the imputed results. MDD: major depressive disorder; GAD: generalised anxiety disorder; OCD: obsessive compulsive disorder; PRS: polygenic risk score.

|  | **Anxiety standardised estimates - *β*_STD_ (95% CIs), *P*** | | | |
| --- | --- | --- | --- | --- |
|  | **Imputed GAD-7  pre-pandemic assessment: Age 22** | **Complete case GAD-7**  **pre-pandemic assessment: Age 22** | **Imputed GAD-7 education adjusted pre-pandemic assessment: Age 22** | **Complete case CISR GAD Symptoms**  **pre-pandemic assessment: Age 24** |
| **Sociodemographic factors** |  |  |  |  |
| Sex (female)  (72%)* | 0.40 (0.33, 0.48) *P* = 3.57 x 10^-23^ | 0.43 (0.33, 0.53) *P* = 4.58 x 10^-17^ | 0.40 (0.32, 0.47) *P* = 2.70 x 10^-22^ | 0.39 (0.30, 0.47) *P* = 8.58 x 10^-20^ |
| Age (older ages)  (scale variable)** | 0.00 (-0.06, 0.07) *P* = 0.945 | -0.01 (-0.09, 0.08) *P* = 0.866 | 0.00 (-0.06, 0.07) *P* = 0.936 | 0.03 (-0.04, 0.10) *P* = 0.377 |
| Lower educational background  (16%) | 0.16 (0.06, 0.26) *P* = 0.002 | 0.12 (-0.01, 0.26) *P* = 0.070 | 0.16 (0.06, 0.27) *P* = 0.002 | 0.11 (0.00, 0.22) *P* = 0.057 |
| Higher income  (scale variable) | -0.04 (-0.07, -0.01) *P* = 0.008 | -0.02 (-0.05, 0.02) *P* = 0.366 | -0.04 (-0.06, -0.01) *P* = 0.017 | -0.03 (-0.06, 0.00) *P* = 0.097 |
| Worse deprivation status (scale variable) | 0.04 (0.01, 0.07) *P* = 0.005 | 0.04 (0.01, 0.08) *P* = 0.034 | 0.04 (0.01, 0.07) *P* = 0.025 | 0.03 (0.00, 0.06) *P* = 0.047 |
| Financial problems  (10%) | 0.24 (0.12, 0.36) *P* = 0.0002 | 0.20 (0.03, 0.36) *P* = 0.021 | 0.22 (0.10, 0.35) *P* = 0.0004 | 0.21 (0.07, 0.34) *P* = 0.002 |
| Partner emotional abuse  (Na) | Not assessed | Not assessed | Not assessed | Not assessed |
| Parent with young children  (11%) | 0.19 (0.08, 0.30) *P* = 0.001 | 0.34 (0.19, 0.48) *P* = 3.61 x 10^-6^ | 0.16 (0.05, 0.27) *P* = 0.004 | 0.21 (0.09, 0.32) *P* = 0.001 |
| **Physical health factors** |  |  |  |  |
| Obesity  (14%) | 0.15 (0.03, 0.27) *P* = 0.012 | 0.11 (-0.03, 0.26) *P* = 0.121 | 0.13 (0.01, 0.25) *P* = 0.039 | 0.16 (0.04, 0.27) *P* = 0.006 |
| Asthma  (10%) | 0.21 (0.07, 0.35) *P* = 0.003 | 0.19 (0.03, 0.26) *P* = 0.018 | 0.20 (0.07, 0.34) *P* = 0.004 | 0.19 (0.06, 0.33) *P* = 0.005 |
| **COVID-19 specific factors** |  |  |  |  |
| COVID-19 infection  (16%) | 0.08 (-0.02, 0.17) *P* = 0.112 | 0.09 (-0.03, 0.21) *P* = 0.145 | 0.08 (-0.02, 0.17) *P* = 0.106 | 0.04 (-0.06, 0.14) *P* = 0.399 |
| Self-isolation  (25%) | 0.17 (0.09, 0.25) *P* = 0.00003 | 0.17 (0.06, 0.27) *P* = 0.002 | 0.17 (0.09, 0.25) *P* = 0.00003 | 0.18 (0.10, 0.27) *P* = 0.00002 |
| Living alone  (6%) | 0.06 (-0.08, 0.21) *P* = 0.392 | -0.04 (-0.23, 0.14) *P* = 0.636 | 0.06 (-0.09, 0.21) *P* = 0.437 | 0.07 (-0.08, 0.23) *P* = 0.340 |
| No access to a garden  (18%) | 0.05 (-0.04, 0.14) *P* = 0.235 | 0.03 (-0.09, 0.14) *P* = 0.642 | 0.06 (-0.03, 0.15) *P* = 0.168 | 0.10 (0.00, 0.19) *P* = 0.050 |
| Health care worker  (12%) | 0.02 (-0.08, 0.13) *P* = 0.652 | 0.03 (-0.11, 0.16) *P* = 0.722 | 0.03 (-0.08, 0.13) *P* = 0.613 | 0.06 (-0.06, 0.17) *P* = 0.337 |
| Key worker  (39%) | 0.02 (-0.05, 0.09) *P* = 0.631 | -0.01 (-0.10, 0.09) *P* = 0.903 | 0.01 (-0.07, 0.08) *P* = 0.894 | 0.02 (-0.05, 0.10) *P* = 0.548 |
| **Psychiatric or mental health factors** |  |  |  |  |
| Probable MDD  (14%) | 0.49 (0.39, 0.62) *P* = 1.33 x 10^-16^ | 0.53 (0.19, 0.66) *P* = 9.83 x 10^-14^ | 0.49 (0.37, 0.60) *P* = 4.06 x 10^-16^ | 0.35 (0.24, 0.47) *P* = 2.36 x 10^-9^ |
| Probable GAD  (13%) | 0.50 (0.39, 0.62) *P* = 2.72 x 10^-17^ | 0.51 (0.37, 0.65) *P* = 6.03 x 10^-12^ | 0.50 (0.39, 0.62) *P* = 3.13 x 10^-16^ | 0.21 (0.07, 0.34) *P* = 0.003 |
| Psychosis like experiences  (15%) | 0.25 (0.15, 0.36) *P* = 4.74 x 10^-6^ | 0.27 (0.14, 0.41) *P* = 0.00006 | 0.24 (0.14, 0.35) *P* = 0.00001 | 0.22 (0.11, 0.32) *P* = 0.00004 |
| Disordered eating  (9%) | 0.26 (0.12, 0.40) *P* = 0.0002 | 0.20 (0.04, 0.37) *P* = 0.014 | 0.25 (0.11, 0.38) *P* = 0.0003 | 0.22 (0.09, 0.35) *P* = 0.001 |
| OCD traits  (scale variable) | 0.15 (0.11, 0.19) *P* = 8.28 x 10^-13^ | 0.17 (0.12, 0.23) *P* = 6.02 x 10^-10^ | 0.15 (0.11, 0.19) *P* = 6.03 x 10^-12^ | 0.15 (0.10, 0.19) *P* = 5.26 x 10^-11^ |
| Autistic traits  (7%) | 0.35 (0.20, 0.51) *P* = 5.18 x 10^-6^ | 0.33 (0.14, 0.52) *P* = 0.001 | 0.34 (0.19, 0.49) *P* = 0.00001 | 0.33 (0.18, 0.49) *P* = 0.00003 |
| Personality disorder traits  (11%) | 0.27 (0.14, 0.40) *P* = 0.00008 | 0.32 (0.17, 0.48) *P* = 0.00006 | 0.26 (0.12, 0.39) *P* = 0.0002 | 0.05 (-0.08, 0.18)† *P* = 0.448 |
| History of alcohol misuse  (9%) | 0.20 (0.07, 0.33) *P* = 0.003 | 0.23 (0.07, 0.39) *P* = 0.004 | 0.20 (0.08, 0.33) *P* = 0.002 | 0.17 (0.05, 0.29) *P* = 0.006 |
| Current smokers (tobacco)  (12%) | 0.10 (-0.01, 0.21) *P* = 0.085 | 0.18 (0.03, 0.33) *P* = 0.019 | 0.08 (-0.03, 0.20) *P* = 0.149 | 0.00 (-0.11, 0.11)† *P* = 0.991 |
| Negative cognitive styles  (scale variable) | 0.07 (0.02, 0.12) *P* = 0.003 | 0.09 (0.03, 0.14) *P* = 0.001 | 0.07 (0.03, 0.12) *P* = 0.003 | 0.09 (0.05, 0.13) *P* = 0.00003 |
| Difficulties accessing mental health info  (23%) | 0.28 (0.19, 0.36) *P* = 1.93 x 10^-9^ | 0.32 (0.21, 0.43) *P* = 1.03 x 10^-8^ | 0.28 (0.19, 0.37) *P* = 9.72 x 10^-10^ | 0.25 (0.16, 0.34) *P* = 1.94 x 10^-7^ |
| Higher neuroticism  (scale variable) | 0.11 (0.07, 0.15) *P* = 7.33 x 10^-7^ | 0.10 (0.05, 0.14) *P* = 0.00009 | 0.11 (0.07, 0.15) *P* = 1.35 x 10^-6^ | 0.11 (0.06, 0.15) *P* = 1.20 x 10^-6^ |
| History of self-harm  (24%) | 0.19 (0.09, 0.28) *P* = 0.0002 | 0.17 (0.07, 0.28) *P* = 0.001 | 0.19 (0.09, 0.29) *P* = 0.0002 | 0.21 (0.12, 0.30) *P* = 3.04 x 10^-6^ |
| Depression PRS  (scale variable) | 0.00 (-0.05, 0.05) *P* = 0.993 | 0.00 (-0.05, 0.05) *P* = 0.993 | 0.00 (-0.05, 0.05) *P* = 0.993 | 0.00 (-0.05, 0.04) *P* = 0.825 |

**Supplement table 12.** Associations between pre-pandemic factors and anxiety during COVID-19 with imputation and complete case data at different pre-pandemic assessments in ALSPAC-young. Results are standardised estimates adjusted for anxiety (at the stated pre-pandemic assessment), sex, age and when the COVID-19 questionnaire was completed. *Indicates the % of individuals with caseness for ALSPAC-parents / ALSPAC-young / GS respectively. **Indicates a continuous scale was used so no proportions are given. †Indicates a substantive difference from the imputed results. MDD: major depressive disorder; GAD: generalised anxiety disorder; OCD: obsessive compulsive disorder; PRS: polygenic risk score.

|  | **Depression and anxiety symptoms standardised estimates - *β*_STD_ (95% CIs), *P*** | |
| --- | --- | --- |
|  | **Depression during COVID-19** | **Anxiety during COVID-19** |
| **Pre-pandemic financial problems at different timings** |  |  |
| At age 27 | 0.20 (0.06, 0.34), *P* = 0.005 | 0.20 (0.03, 0.36), *P* = 0.021 |
| At age 26 | 0.21 (0.09, 0.33), *P* = 0.0004 | 0.34 (0.19, 0.49), *P* = 6.66 x 10^-6^ |
| At age 25 | 0.17 (0.04, 0.30), *P* = 0.01 | 0.22 (0.06, 0.37), *P* = 0.005 |
| At age 24 | 0.20 (0.08, 0.34), *P* = 0.002 | 0.50 (0.35, 0.66), *P* = 2.45 x 10^-10^ |

**Supplement table 13.** Associations between financial problems and depression and anxiety symptoms during COVID-19 with different pre-pandemic timings of financial problems in ALSPAC-young, using complete case data. Results are standardised estimates adjusted for pre-pandemic depression and anxiety (the most recent timing of depression and anxiety available), sex, age and when the COVID-19 questionnaire was completed. The same criteria were used for deriving financial problems at each age, see supplement methods for more details.

|  | **Depression odds ratio (95% CIs), *P*** | | | **Anxiety odds ratio (95% CIs), *P*** | | |
| --- | --- | --- | --- | --- | --- | --- |
|  | **ALSPAC-parents** | **ALSPAC-young** | **Gen Scot** | **ALSPAC-parents** | **ALSPAC-young** | **Gen Scot** |
| **Sociodemographic factors** |  |  |  |  |  |  |
| Sex (female)  (76% / 72% / 64%)* | 1.94 (1.04, 3.62) *P* = 0.036  (n=2789) | 2.23 (1.63, 3.07) *P* = 6.91 x 10^-7^  (n=2219) | 1.98 (1.47, 2.66) *P* = 6.11 x 10^-6^  (n=3108) | 3.79 (2.35, 6.12) *P* = 4.77 x 10^-8^  (n=3052) | 2.69 (1.96, 3.69) *P* = 7.45 x 10^-10^  (n=1811) | 1.57 (1.18, 2.09) *P* = 0.002  (n=3099) |
| Age (older ages)  (scale variable)** | 0.92 (0.88, 0.97) *P* = 0.001  (n=2789) | 0.89 (0.71, 1.11) *P* = 0.293  (n=2219) | 0.96 (0.95, .97) *P* = 2.00 x 10^-16^  (n=3108) | 0.93 (0.90, 0.95) *P* = 1.70 x 10^-7^  (n=3052) | 1.02 (0.82, 1.26) *P* = 0.876  (n=1811) | 0.96 (0.95, .97) *P* = 1.91 x 10^-13^  (n=3099) |
| Lower educational background  (11%/ 16% / 13%) | 1.89 (1.07, 3.34) *P* = 0.029  (n=2635) | 1.5 (1.07, 2.13) *P* = 0.020  (n=2008) | 1.25 (0.88, 1.78) *P* = 0.211  (n=2934) | 1.55 (1.00, 1.78) *P* = 0.024  (n=2945) | 1.26 (0.89, 1.78) *P* = 0.200  (n=1685) | 1.42 (0.96, 2.10) *P* = 0.080  (n=2926) |
| Higher income  (scale variable) | 0.85 (0.78, 0.91) *P* = 0.00002  (n=2386) | 0.92 (0.83, 1.01) *P* = 0.082  (n=2068) | 0.73 (0.65, 0.82) *P* = 3.95 x 10^-7^  (n=2748) | 0.93 (0.88, 0.98) *P* = 0.007  (n=2179) | 0.91 (0.83, 1.01) *P* = 0.072  (n=1491) | 0.84 (0.75, 0.95) *P* = 0.004  (n=2741) |
| Worse deprivation status (scale variable) | 1.20 (1.02, 1.42) *P* = 0.026  (n=2627) | 1.04 (0.94, 1.14) *P* = 0.469  (n=2057) | 1.13 (1.02, 1.24) *P* = 0.017  (n=2956) | 1.19 (1.07, 1.32) *P* = 0.001  (n=2872) | 1.12 (1.01, 1.23) *P* = 0.024  (n=1701) | 1.10 (0.99, 1.21) *P* = 0.067  (n=2948) |
| Financial problems  (11% / 10% / 4%) | 2.32 (1.37, 3.94) *P* = 0.002  (n=2379) | 2.09 (1.47, 2.99) *P* = 4.63 x 10^-5^  (n=2035) | 1.95 (1.19, 3.18) *P* = 0.008  (n=3087) | 1.95 (1.32, 2.87) *P* = 0.001  (n=2356) | 1.74 (1.17, 2.59) *P* = 0.006  (n=1639) | 1.73 (1.07, 2.79) *P* = 0.025  (n=3077) |
| Partner emotional abuse  (8% / Na / Na) | 2.67 (1.50, 4.73) *P* = 0.001  (n=2371) | Not assessed | Not assessed | 2.69 (1.77, 4.08) *P* = 3.49 x 10^-6^  (n=2344) | Not assessed | Not assessed |
| Parent with young children  (Na / 11% / 11%) | Not assessed | 1.18 (0.83, 1.68) *P* = 0.345  (n=2217) | 0.93 (0.62, 1.38) *P* = 0.710  (n=3108) | Not assessed | 1.94 (1.39, 2.70) *P* = 8.70 x 10^-5^  (n=1809) | 1.20 (0.82, 1.76) *P* = 0.345  (n=3099) |
| **Physical health factors** |  |  |  |  |  |  |
| Obesity  (18 % / 14% / 20%) | 2.89 (1.65, 5.05) *P* = 0.0002  (n=2474) | 1.51 (1.05, 2.16) *P* = 0.025  (n=1786) | 3.18 (2.30, 4.40) *P* = 2.57 x 10^-12^  (n=2957) | 1.48 (1.01, 2.15) *P* = 0.042  (n=2470) | 1.38 (0.96, 1.97) *P* = 0.081  (n=1488) | 1.62 (1.17, 2.23) *P* = 0.003  (n=2950) |
| Asthma  (16% / 10% / 10%) | 1.13 (0.63, 2.02) *P* = 0.677  (n=2552) | 1.36 (0.89, 2.07) *P* = 0.159  (n=1809) | 1.29 (0.89, 1.87) *P* = 0.181  (n=3071) | 1.46 (1.05, 2.03) *P* = 0.023  (n=2932) | 1.33 (0.90, 1.97) *P* = 0.158  (n=1631) | 1.43 (0.99, 2.07) *P* = 0.056  (n=3071) |
| **COVID-19 specific factors** |  |  |  |  |  |  |
| COVID-19 infection  (12% /16% / 8%) | 1.77 (1.07, 2.95) *P* = 0.027  (n=2772) | 1.18 (0.86, 1.63) *P* = 0.303  (n=2215) | 1.62 (1.05, 2.50) *P* = 0.031  (n=3107) | 1.64 (1.17, 2.29) *P* = 0.004  (n=3030) | 1.17 (0.86, 1.59) *P* = 0.322  (n=1809) | 1.30 (0.84, 2.01) *P* = 0.247  (n=3098) |
| Self-isolation  (19% /25% / Na) | 2.15 (1.38, 3.36) *P* = 0.001  (n=2757) | 1.42 (1.09, 1.85) *P* = 0.010  (n=2197) | Not assessed | 1.61 (1.21, 2.15) *P* = 0.001  (n=3021) | 1.62 (1.25, 2.09) *P* = 0.0002  (n=1797) | Not assessed |
| Living alone  (8% / 6% / 16%) | 3.40 (1.97, 6.19) *P* = 0.00006  (n=2780) | 1.14 (0.71, 1.84) *P* = 0.583  (n=2207) | 1.71 (1.26, 2.31) *P* = 0.001  (n=3106) | 0.96 (0.59, 1.56) *P* = 0.877  (n=3036) | 0.87 (0.53, 1.44) *P* = 0.602  (n=1802) | 1.04 (0.75, 1.45) *P* = 0.809  (n=3097) |
| No access to a garden  (2% /18% / 8%) | 1.85 (0.52, 6.63) *P* = 0.343  (n=2775) | 1.26 (0.93, 1.71) *P* = 0.141  (n=2208) | 2.15 (1.48, 3.12) *P* = 0.00006  (n=3105) | 1.43 (0.61, 3.36) *P* = 0.414  (n=3030) | 0.99 (0.73, 1.35) *P* = 0.976  (n=1800) | 1.55 (1.04, 2.32) *P* = 0.031  (n=3096) |
| Health care worker  (11% / 12% / NA) | 0.73 (0.35, 1.50) *P* = 0.389  (n=2767) | 0.88 (0.61, 1.27) *P* = 0.496  (n=2196) | Not assessed | 0.72 (0.48, 1.09) *P* = 0.120  (n=3024) | 0.97 (0.69, 1.38) *P* = 0.874  (n=1792) | Not assessed |
| Key worker  (32% / 39% / 22%) | 1.15 (0.75, 1.76) *P* = 0.529  (n=2719) | 0.74 (0.57, 0.95) *P* = 0.018  (n=2155) | 0.86 (0.63, 1.16) *P* = 0.317  (n=3105) | 1.02 (0.78, 1.34) *P* = 0.884  (n=2964) | 0.97 (0.76, 1.23) *P* = 0.780  (n=1765) | 1.18 (0.88, 1.56) *P* = 0.257  (n=3096) |
| **Psychiatric or mental health factors** |  |  |  |  |  |  |
| Probable MDD  (8% / 14% / 14%) | 2.57 (1.50, 4.41) *P* = 0.001  (n=2545) | 2.05 (1.49, 2.80) *P* = 8.04 x 10^-6^  (n=1969) | 2.47 (1.80, 3.38) *P* = 1.81 x 10^-8^  (n=2816) | 1.74 (1.18, 2.55) *P* = 0.005  (n=2924) | 2.92 (2.14, 3.99) *P* = 1.7 x 10^-11^  (n=1649) | 2.05 (1.50, 2.80) *P* = 7.31 x 10^-6^  (n=2810) |
| Probable GAD  (7% / 13% / Na) | 1.96 (1.12, 3.43) *P* = 0.019  (n=2596) | 1.56 (1.12, 2.16) *P* = 0.008  (n=1969) | Not assessed | 1.60 (1.08, 2.36) *P* = 0.020  (n=2967) | 2.75 (1.99, 3.81) *P* = 1.12 x 10^-9^  (n=1648) | Not assessed |
| Psychosis like experiences  (Na / 15% / scale) | Not assessed | 1.73 (1.26, 2.37) *P* = 0.001  (n=1967) | 1.39 (1.20, 1.63) *P* = 0.00002  (n=1820) | Not assessed | 1.65 (1.20, 2.28) *P* = 0.002  (n=1644) | 1.36 (1.18, 1.58) *P* = 0.00004  (n=1816) |
| Disordered eating  (3% / 9% / Na) | 0.71 (0.25, 2.04) *P* = 0.523  (n=2558) | 1.82 (1.24, 2.67) *P* = 0.002  (n=1932) | Not assessed | 1.58 (1.90, 2.78) *P* = 0.114  (n=2938) | 1.86 (1.27, 2.72) *P* = 0.001  (n=1589) | Not assessed |
| OCD traits  (scale variable) | Not assessed | 1.18 (1.04, 1.33) *P* = 0.010  (n=1964) | Not assessed | Not assessed | 1.46 (1.28, 1.67) *P* = 2.36 x 10^-8^  (n=1617) | Not assessed |
| Autistic traits  (Na / 7% / Na) | Not assessed | 1.89 (1.25, 2.87) *P* = 0.003  (n=1964) | Not assessed | Not assessed | 1.49 (0.92, 2.43) *P* = 0.107  (n=1617) | Not assessed |
| Personality disorder traits  (11% /11% / Na) | 2.16 (1.31, 3.56) *P* = 0.002  (n=2424) | 1.48 (1.01, 2.16) *P* = 0.042  (n=1762) | Not assessed | 1.45 (1.02, 2.06) *P* = 0.040  (n=2805) | 1.94 (1.33, 2.83) *P* = 0.001  (n=1474) | Not assessed |
| History of alcohol misuse  (17% /9% / 16%) | 1.34 (0.82, 2.20) *P* = 0.245  (n=2764) | 1.41 (0.94, 2.12) *P* = 0.093  (n=1940) | 1.01 (0.69, 1.48) *P* = 0.949  (n=2816) | 1.05 (0.74, 1.51) *P* = 0.779  (n=2670) | 1.86 (1.26, 2.75) *P* = 0.002  (n=1625) | 0.94 (0.63, 1.41) *P* = 0.767  (n=2806) |
| Current smokers (tobacco)  (29%/ 12% /10%) | 1.48 (0.97, 2.26) *P* = 0.070  (n=2765) | 1.10 (0.77, 1.59) *P* = 0.595  (n=1960) | 1.79 (1.26, 2.55) *P* = 0.001  (n=3056) | 1.44 (1.09, 1.92) *P* = 0.012  (n=2672) | 1.84 (1.28, 2.65) *P* = 0.001  (n=1634) | 1.50 (1.05, 2.15) *P* = 0.025  (n=3047) |
| Negative cognitive styles  (scale variable) | 1.85 (1.49, 2.30) *P* = 2.01 x 10^-8^  (n=2781) | 1.41 (1.22, 1.63) *P* = 5.28 x 10^-6^  (n=1445) | 1.77 (1.57, 1.99) *P* = 2.00 x 10^-16^  (n=3078) | 1.47 (1.28, 1.68) *P* = 2.70 x 10^-8^  (n=2665) | 1.23 (1.07, 1.40) *P* = 0.003  (n=1284) | 1.52 (1.34, 1.71) *P* = 1.05 x 10^-11^  (n=3069) |
| Difficulties accessing mental health info  (Na / 23% / Na) | Not assessed | 1.31 (1.00, 1.71) *P* = 0.046  (n=2198) | Not assessed | Not assessed | 2.13 (1.62, 2.81) *P* = 5.61 x 10^-8^  (n=1601) | Not assessed |
| Higher neuroticism  (scale variable) | Not assessed | 1.18 (1.02, 1.36) *P* = 0.031  (n=1587) | 1.69 (1.50, 1.91) *P* = 2.00 x 10^-16^  (n=2965) | Not assessed | 1.30 (1.13, 1.49) *P* = 0.0002  (n=1397) | 1.73 (1.52, 1.97) *P* = 2.00 x 10^-16^  (n=2965) |
| History of self-harm  (Na / 24% / 2%) | Not assessed | 1.71 (1.29, 2.25) *P* = 0.0002  (n=1972) | 4.26 (1.80, 10.10) *P* = 0.001  (n=2861) | Not assessed | 1.70 (1.30, 2.22) *P* = 0.0001  (n=1651) | 3.01 (1.33, 6.82) *P* = 0.008  (n=2853) |
| Depression PRS  (scale variable) | 1.53 (1.18, 1.98) *P* = 0.001  (n=1906) | 1.01 (0.88, 1.17) *P* = 0.836  (n=1592) | 1.15 (1.01, 1.31) *P* = 0.041  (n=2861) | 1.31 (1.13, 1.53) *P* = 0.0004  (n=2071) | 1.10 (0.96, 1.26) *P* = 0.174  (n=1329) | 1.14 (1.01, 1.30) *P* = 0.037  (n=2853) |

**Supplement table 14.** Logistic regressions between pre-pandemic factors and depression and anxiety during COVID-19 using complete case data in all cohorts. Results are odds ratios adjusted for depression or anxiety, sex, age and when the COVID-19 questionnaire was completed. *Indicates the % of individuals with caseness in ALSPAC-parents / ALSPAC-young / GS respectively. **Indicates a continuous scale was used. N’s vary due to missing data. MDD: major depressive disorder; GAD: generalised anxiety disorder; OCD: obsessive compulsive disorder; PRS: polygenic risk score. Baseline data was continuous in GS as no thresholds exist for the sub scales of GHQ-28.
